# Spatiotemporally Controlled Bioorthogonal Prodrug Activation for Precise Chemotherapy

**DOI:** 10.1002/smsc.202500483

**Published:** 2025-11-21

**Authors:** Xia Liu, Xiao Liang, Ziqi Fang, Fan Liu, Wenbin Zhong, Yiqun Wan, Hao Wan

**Affiliations:** ^1^ College of Chemistry Fuzhou University Fuzhou 350108 P. R. China; ^2^ State Key Laboratory of Food Science and Resources Nanchang University Nanchang 330047 P. R. China; ^3^ School of Chemistry and Chemical Engineering Nanchang University Nanchang 330031 P. R. China

**Keywords:** bioorthogonal reaction, nanoplatform, precise chemotherapy, prodrug activation, spatiotemporal control

## Abstract

The uncontrolled pharmacokinetics of anticancer drugs after systemic administration can cause off‐target accumulation in healthy tissues, compromising the antitumor efficacy and posing serious safety issues. To address these limitations, the spatiotemporally controlled inverse electron demand Diels–Alder reaction (SC‐IEDDA) strategy is developed, which controls bioorthogonal IEDDA reactions within tumor tissues for in situ prodrug activation and precise chemotherapy. The strategy employs two nanoplatforms: 1) pH‐sensitive zeolitic imidazolate framework‐8 (ZIF‐8) nanoparticles encapsulating *trans*‐cyclooctene‐caged doxorubicin (TCO‐DOX, the prodrug) and 2) indocyanine green (ICG)‐loaded near‐infrared (NIR) light‐responsive nanomicelles constructed from an amphiphilic molecule comprising the tetrazine (Tz) moiety conjugated to polyethylene glycol via a thioketal (TK) linker. During systemic circulation, both nanoplatforms remain intact to prevent premature prodrug activation. Following tumor accumulation via the enhanced permeability and retention effect, the acidic environment triggers ZIF‐8 degradation, locally releasing TCO‐DOX. Simultaneously, NIR laser irradiation induces ICG's production of reactive oxygen species, cleaving the TK linker to liberate the Tz activator. This enables the precise triggering of bioorthogonal IEDDA reaction between TCO‐DOX and Tz at the tumor site, ensuring the uncaging of doxorubicin to exert efficient antitumor efficacy. This strategy represents a critical advancement in the safe and effective application in precision oncology.

## Graphical abstract

A spatiotemporally controlled prodrug activation strategy is developed by integrating bioorthogonal inverse electron demand Diels–Alder reaction with stimulus‐responsive nanoplatforms. Following intravenous injection, the two nanoplatforms independently respond to low pH and near‐infrared light irradiation, releasing the prodrug and liberating the activator. This enables in situ activation of the prodrug at the tumor site for precise chemotherapy.

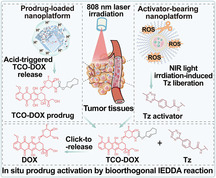

## 1 Introduction

Bioorthogonal reaction, which enables chemical transformations in biological environments with minimal interference to physiological processes,^[^ ^1^, ^2^, ^3^ ^]^ has emerged as a powerful tool for localized activation of therapeutic agents in vivo to combat cancer,^[^ ^4^, ^5^ ^]^ precisely exerting cytotoxicity at the tumor site and minimizing potential damage to normal tissues.^[^ ^6^, ^7^, ^8^ ^]^ A variety of bioorthogonal reactions have been explored for in situ drug synthesis in vivo, including Staudinger ligation,^[^ ^9^ ^]^ metal‐catalyzed transformations,^[^ ^10^, ^11^, ^12^, ^13^ ^]^ and inverse electron‐demand Diels–Alder (IEDDA) reaction.^[^ ^14^, ^15^, ^16^, ^17^, ^18^, ^19^ ^]^ Among these, the IEDDA‐mediated “click‐to‐release” reaction between *trans*‐cyclooctene (TCO) and tetrazine (Tz) stands out due to its exceptional chemoselectivity, rapid kinetics, and modular reactivity.^[^ ^20^, ^21^, ^22^, ^23^, ^24^ ^]^ Ideally, TCO‐caged prodrugs remain stable during systemic circulation and become activated by reacting with Tz‐based moieties at the tumor site. However, poor tumor‐to‐normal tissue accumulation preference severely limits their tumor targeting specificity.^[^ ^25^, ^26^ ^]^ Moreover, mismatched pharmacokinetics between Tz‐based activators and TCO‐caged prodrugs always result in stochastic interactions, significantly compromising the antitumor efficacy and leading to unintended prodrug activation in healthy tissues to cause damage.^[^ ^27^, ^28^, ^29^, ^30^, ^31^ ^]^ Thus, new strategies are urgently needed to achieve spatiotemporal control over prodrug activation precisely at the tumor site for the safe and effective antitumor process.

Nanoparticle‐based drug delivery systems (DDSs) have gained considerable attention due to their potential to achieve controlled drug release and improve drug pharmacokinetics.^[^ ^32^, ^33^, ^34^ ^]^ Stimuli‐responsive DDSs, which respond to internal (e.g., pH and redox state) or external (e.g., light and heat) triggers, offer significant promise for enhancing therapeutic efficacy and precision.^[^ ^35^ ^]^ However, DDSs that rely on a single stimulus often fall short in complex biological environments. Internally triggered DDSs, in particular, are challenged by tumor heterogeneity and the presence of overlapping microenvironmental features between tumor and healthy tissues.^[^ ^36^ ^]^ These factors may result in unpredictable and uncontrollable drug release profiles, increasing the risk of off‐target effects and systemic toxicity.^[^ ^37^, ^38^ ^]^ While DDSs responding solely to external stimuli provide the temporal control, they often fail to achieve site‐specific drug release and may cause nonspecific damage to peripheral tissues.^[^ ^39^, ^40^ ^]^ Therefore, the responsive DDS‐based strategy integrating both endogenous and exogenous stimuli is beneficial for enhancing the precision and safety of drug delivery.

**Scheme 1:**
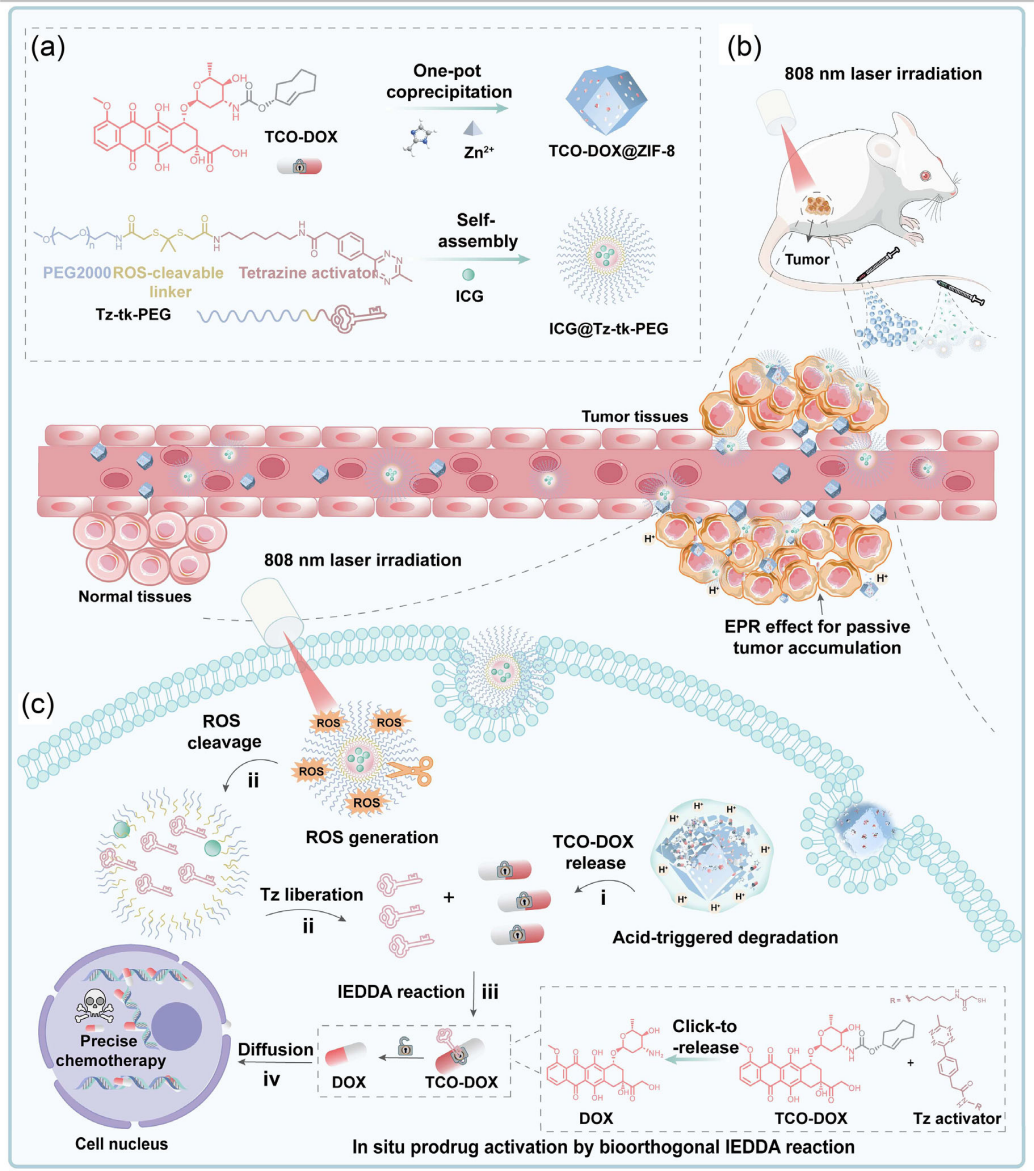
Schematic illustration of the SC‐IEDDA strategy for spatiotemporally controlled bioorthogonal prodrug activation at the tumor site. a) The fabrication processes of the two nanoplatforms: TCO‐DOX@ZIF‐8 and ICG@Tz‐tk‐PEG. b) The in vivo delivery of the nanoplatforms, their tumor accumulation via the enhanced permeability and retention (EPR) effect, and the subsequent NIR laser irradiation that triggers the cascade reactions. c) The key stimuli‐responsive release processes and cascade reaction within the tumor: (i) acidic degradation of ZIF‐8 to release TCO‐DOX, (ii) NIR‐induced liberation of Tz activator from ICG@Tz‐tk‐PEG, (iii) the spontaneous IEDDA reaction between TCO‐DOX and Tz moiety for tumor‐localized DOX generation, and (iv) diffusion of activated DOX into the nucleus to induce cell death.

Herein, we propose a novel strategy, termed SC‐IEDDA, which integrates a biorthogonal IEDDA reaction with nanoplatforms independently responsive to endogenous and exogenous stimuli. This strategy is designed to enable in situ prodrug activation at the tumor site with spatiotemporal precision for effective chemotherapy against cancer (**Scheme** **1**). Specifically, zeolitic imidazolate framework‐8 (ZIF‐8) is employed as a pH‐sensitive nanocarrier to encapsulate doxorubicin (DOX) in its TCO‐caged prodrug form (TCO‐DOX), yielding TCO‐DOX@ZIF‐8. In parallel, a near‐infrared (NIR) light‐responsive nanomicelle (ICG@Tz‐tk‐PEG) loaded with indocyanine green (ICG, a photodynamic agent that has been approved for clinical utilization^[^ ^41^ ^]^) is developed, which is constructed by the self‐assembly of an amphiphilic molecule containing a tetrazine (Tz) moiety conjugated to polyethylene glycol (PEG) via a reactive oxygen species (ROS)‐cleavable thioketal (TK) linker.^[^ ^42^ ^]^ Following intravenous coadministration, both nanoplatforms are expected to maintain systemic circulation without premature prodrug activation, owing to the shielding effects of the ZIF‐8 structure and the hydrophilic PEG corona, which prevent unintended interactions between Tz and TCO‐DOX. Upon passive accumulation at the tumor site via the enhanced permeability and retention (EPR) effect,^[^ ^43^, ^44^ ^]^ ZIF‐8 is anticipated to disassemble in the acidic microenvironment, releasing TCO‐DOX locally. Simultaneously, NIR laser irradiation is applied to trigger ROS generation, cleaving the TK linker and liberating Tz for in situ bioorthogonal activation of TCO‐DOX via a spontaneous IEDDA reaction. This cascaded design, which combines bioorthogonal chemistry with stimuli‐responsive nanotechnology, aims to achieve potent chemotherapy while minimizing off‐target effects, thereby offering a promising platform for advancing precision oncology.

## 2 Results and Discussion

### 2.1 Preparation and Characterization of TCO‐DOX@ZIF‐8 and ICG@Tz‐tk‐PEG

**Figure 1:**
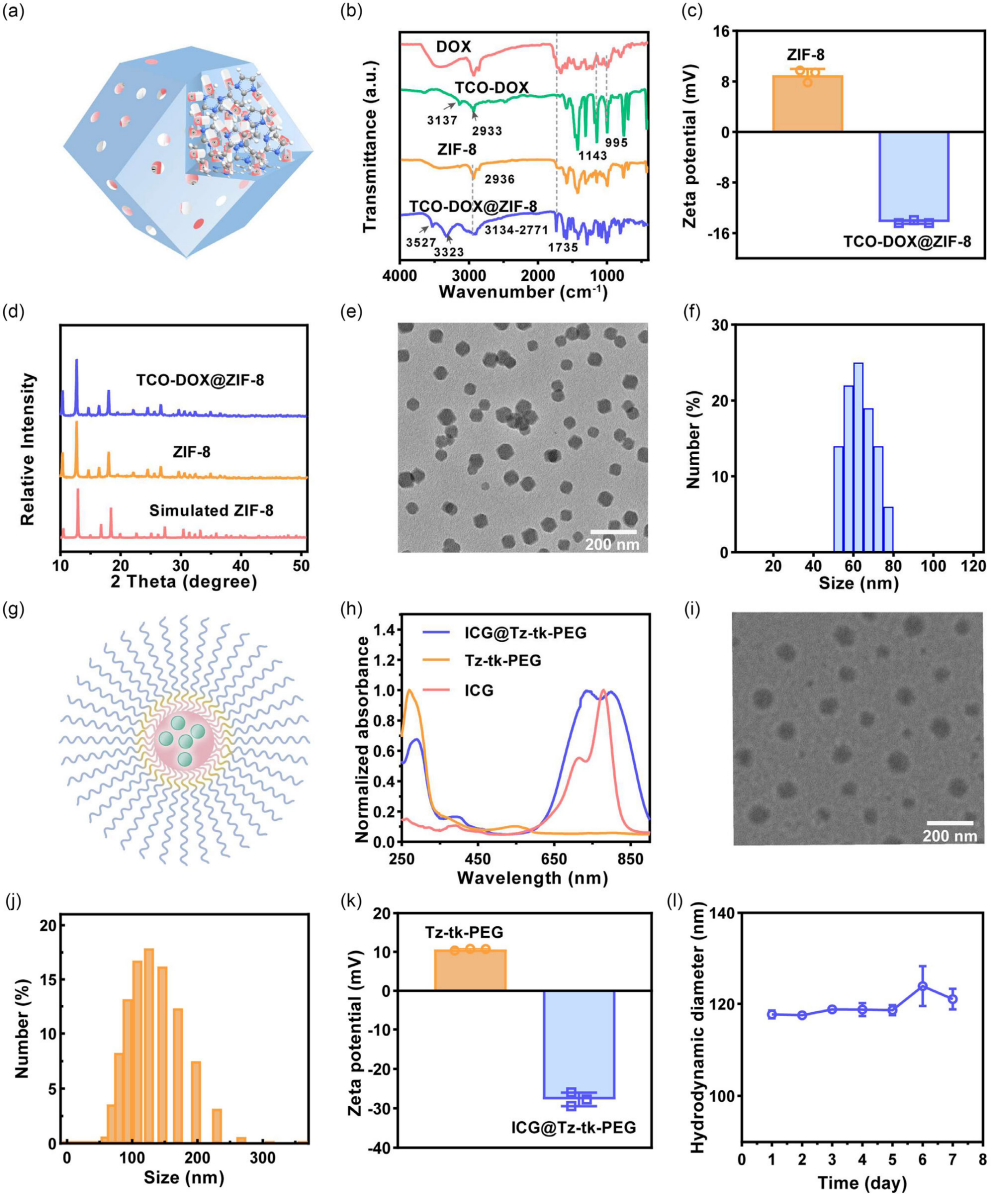
Characterization of TCO‐DOX@ZIF‐8 and ICG@Tz‐tk‐PEG. a) Schematic representation of the TCO‐DOX@ZIF‐8 nanoplatform. b) FTIR spectra of DOX, TCO‐DOX, ZIF‐8, and TCO‐DOX@ZIF‐8. c) Zeta potential analysis of ZIF‐8 and TCO‐DOX@ZIF‐8. d) XRD patterns of simulated ZIF‐8, synthesized ZIF‐8, and TCO‐DOX@ZIF‐8. e) TEM image of TCO‐DOX@ZIF‐8. Scale bar = 200 nm. f) Size distribution of TCO‐DOX@ZIF‐8. g) Schematic representation of the ICG@Tz‐tk‐PEG nanomicelle. h) UV‐vis absorption spectra of ICG, Tz‐tk‐PEG, and ICG@Tz‐tk‐PEG. i) TEM image of ICG@Tz‐tk‐PEG. Scale bar = 200 nm. j) Size distribution of ICG@Tz‐tk‐PEG. k) Zeta potentials of Tz‐tk‐PEG and ICG@Tz‐tk‐PEG. l) Size variation of ICG@Tz‐tk‐PEG in phosphate‐buffered saline (PBS) over 7 days.

The chemotherapeutic agent DOX is extensively utilized in the treatment of diverse malignancies. However, its nonselective cytotoxicity toward both cancerous and healthy cells results in strict dose‐limiting utilization and severe side effects, greatly limiting clinical utility.^[^ ^45^ ^]^ To address these limitations, we proposed the SC‐IEDDA strategy. Here, we first synthesized the prodrug TCO‐DOX by conjugating the amino group of DOX to TCO (please see detailed synthesis processes in Supporting Information), and the structure was confirmed via detailed analyses of ^1^H nuclear magnetic resonance (NMR), ^13^C NMR, and high‐resolution mass spectrometry (HR‐MS) (Figure S1–S4, Supporting Information). The resulting TCO‐DOX was then encapsulated into ZIF‐8 nanoparticles via a one‐pot coprecipitation process (**Figure** **1**a, please see detailed fabrication processes in Supporting Information), obtaining the TCO‐DOX@ZIF‐8 nanoplatform with a TCO‐DOX loading capacity (LC) of up to 25.2%. Fourier transform infrared (FT‐IR) spectroscopy analysis clearly illustrated the structural transformation during TCO‐DOX synthesis and indicated its successful encapsulation within ZIF‐8 nanoparticles (Figure 1b). Specifically, TCO‐DOX, distinct from pristine DOX, showed characteristic bands at 1143 cm^−1^ and 995 cm^−1^, corresponding to urethane carbamate C—O stretching and amide C—N stretching, respectively. Its subsequent encapsulation by ZIF‐8 induced pronounced band broadening within 3134–2711 cm^−1^ (C—H···π interactions) and a 390 cm^−1^ shift of hydroxyl stretches (from 3137–2933 cm^−1^ to 3527–3323 cm^−1^), suggesting confinement‐driven hydrogen bonding. Zeta potential analysis showed a shift from + 8.98 mV for bare ZIF‐8 to −14.25 mV for TCO‐DOX@ZIF‐8 (Figure 1c), further evidencing the successful prodrug loading. As disclosed by X‐ray diffraction (XRD) analysis, the crystalline structure of TCO‐DOX@ZIF‐8 well matched that of pristine ZIF‐8 (Figure 1d), suggesting that the loading process didn't alter the intrinsic framework, crucial for preserving the pH‐sensitive property of ZIF‐8.^[^ ^46^ ^]^ Transmission electron microscopy (TEM) observations directly confirmed that the as‐prepared TCO‐DOX@ZIF‐8 particles exhibit a hexagonal morphology and good monodispersity, with an average diameter of 62.1 ± 0.44 nm (Figure 1e,f). Additionally, no obvious change in particle size was detected over 36 h under physiological conditions (Figure S21, Supporting Information), making this material suitable for passive tumor drug delivery via the EPR effect.^[^ ^47^, ^48^, ^49^ ^]^

To serve as a complementary activator for controlled activation of TCO‐DOX, we synthesized an amphiphilic molecule (Tz‐tk‐PEG) bearing the Tz activator via a five‐step synthetic route (please see detailed synthesis processes in Supporting Information). As illustrated in Figure 1g, Tz‐tk‐PEG consists of a hydrophilic PEG chain, a ROS‐cleavable TK linker, and a hydrophobic Tz segment. Structural validation of intermediates and final products using ^1^H NMR, ^13^C NMR, and HR‐MS confirmed the successful synthesis of Tz‐tk‐PEG (Figure S5–S20, Supporting Information). The critical micelle concentration (CMC) for Tz‐tk‐PEG was determined to be 78.4 μg mL^−1^ by the fluorescence probe method (Figure S22, Supporting Information).^[^ ^50^ ^]^ UV‐visible (UV‐Vis) absorption spectra revealed red‐shifted peaks from 271 nm (Tz‐tk‐PEG) to 288 nm and from 780 nm (ICG) to 800 nm, along with spectral broadening around 780 nm, upon the codissolution of ICG and Tz‐tk‐PEG into an aqueous environment (Figure 1h). These shifts are attributed to π–π stacking interactions between the *p*‐phenyltetrazine moiety of Tz‐tk‐PEG and the indole ring of ICG, suggesting ICG's involvement in micellar self‐assembly and thus guaranteeing the premise for subsequent NIR laser irradiation‐induced ROS production by ICG for TK cleavage to liberate Tz. To minimize the phototoxicity, the LC of ICG was optimized to be 5 wt% (Figure S23 and S24, Supporting Information). Representative TEM image of ICG@Tz‐tk‐PEG clearly verified the formation of monodispersed spherical micelles with a diameter centered at 105.88 nm (Figure 1i), which is in good agreement with the narrow hydrodynamic diameter distribution profiled by dynamic light scattering (DLS) analysis (Figure 1j). The size feature supports the passive targeting of tumors due to the EPR effect.^[^ ^51^, ^52^ ^]^ Furthermore, the ICG@Tz‐tk‐PEG nanoplatform demonstrated remarkable colloidal stability with a zeta potential of ≈−27 mV (Figure 1k,l), suitable for biological applications.^[^ ^53^, ^54^ ^]^

### 2.2 pH/NIR Light‐Responsive Prodrug Activation Profiles

**Figure 2:**
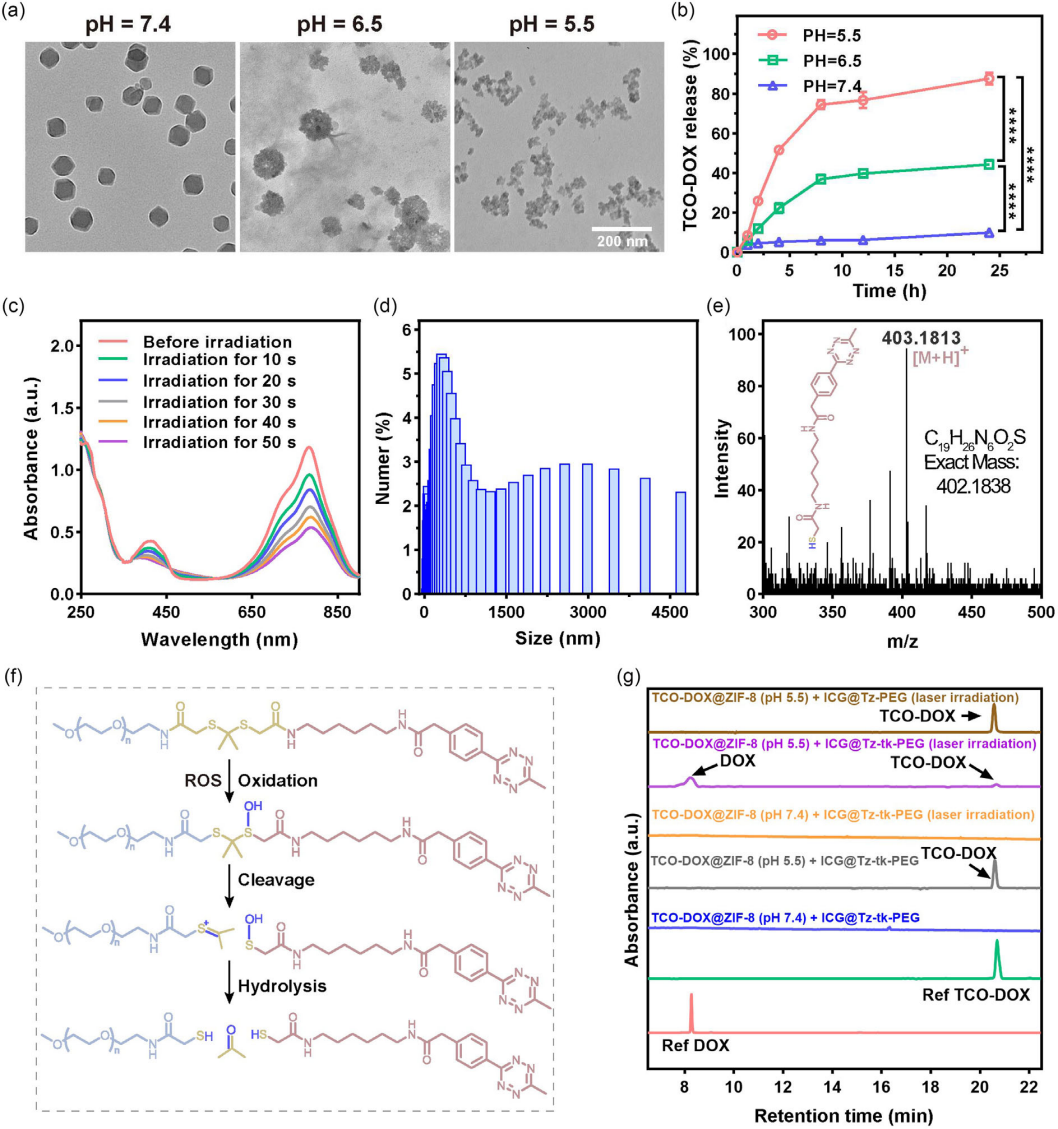
The stimulus‐responsive behaviors of TCO‐DOX@ZIF‐8 and ICG@Tz‐tk‐PEG. a) TEM images of TCO‐DOX@ZIF‐8 dispersed in PBS at pH 5.5, 6.5, or 7.4 for 6 h. Scale bar = 200 nm. b) TCO‐DOX release profiles from TCO‐DOX@ZIF‐8 in PBS solution with different pH values (i.e., pH 5.5, 6.5, and 7.4) (*n* = 3 independent experiments). c) ROS generation by ICG@Tz‐tk‐PEG under 808 nm laser irradiation, as measured by the ROS indicator 1,3‐diphenylisobenzofuran (DPBF). d) DLS analysis of ICG@Tz‐tk‐PEG following irradiation with an 808 nm laser (0.8 W cm^−2^, 2 min). e) HR‐MS analysis of ICG@Tz‐tk‐PEG fragmentation initiated by 808 nm laser irradiation (0.8 W cm^−2^, 2 min). f) Proposed oxidative degradation mechanism of Tz‐tk‐PEG. g) High‐performance liquid chromatography (HPLC) analysis of TCO‐DOX activation under different conditions (detected at 450 nm). Data points were presented as mean ± s.d. Statistical analysis was calculated via one‐way ANOVA with a Tukey post hoc test. ****P < 0.0001 represents a significant difference.

After constructing the two nanoplatforms, we initially evaluated the pH‐responsive properties of TCO‐DOX@ZIF‐8. TEM analysis intuitively demonstrated that TCO‐DOX@ZIF‐8 retained its structural integrity at normal physiological pH (i.e., ≈7.4). In contrast, exposure to acidic conditions, such as the tumor microenvironment (i.e., pH ≈ 6.5) and endo/lysosomal (i.e., pH ≈ 5.5) environment where most nanoplatforms reside after phagocytosis by tumor cells,^[^ ^55^ ^]^ led to progressive degradation, confirming acid‐triggered destabilization of the ZIF‐8 structure (**Figure** **2**a). Enlightened by this, we next investigated the pH‐responsive release profile of TCO‐DOX from TCO‐DOX@ZIF‐8. As shown in Figure 2b, less than 10% of the prodrug was released at pH 7.4 within 24 h, indicating minimal leakage under normal physiological conditions. However, at pH 6.5 and pH 5.5, the release increased significantly, reaching ≈ 44% and ≈88%, respectively, over the same period. These results strongly suggest that TCO‐DOX@ZIF‐8 can effectively reduce premature prodrug leakage during blood circulation after systemic administration while enabling efficient release within the acidic environment, that is, the tumor. Parallel evaluation of the NIR light‐responsiveness of ICG@Tz‐tk‐PEG revealed that 808 nm laser irradiation for short periods (0.8 W cm^−2^) induced the substantial production of ROS (Figure 2c), attributed to the photodynamic property of ICG. This led to the micellar disassembly after only 2 min laser irradiation, as evidenced by the appearance of irregularly shaped particles ranging from 0 to 4000 nm (Figure 2d and S25, Supporting Information). As a result, mass spectrometry analysis of the irradiated fragments (Figure 2e) identified a characteristic peak at m/z = 403.1819 ([M+H]^+^), corresponding to the liberated Tz moiety. Notably, the correlation between the time‐dependent increase in this signal and the ROS generation profile (Figure S26, Supporting Information) confirms that the release is driven by ROS. These findings support a mechanism wherein ICG, acting as a photosensitizer, produces ROS under NIR light, which cleaves the TK linker. This rapidly triggers micellar disassembly and liberates Tz molecules (Figure 2f),^[^ ^56^ ^]^ guaranteeing the premise of temporal control.

With the above pH‐ and NIR light‐responsive behaviors fully characterized, we eventually moved to examine the activation of TCO‐DOX under four conditions: (1) TCO‐DOX@ZIF‐8 (pH 7.4) + ICG@Tz‐tk‐PEG; (2) TCO‐DOX@ZIF‐8 (pH 5.5) + ICG@Tz‐tk‐PEG; (3) TCO‐DOX@ZIF‐8 (pH 7.4) + ICG@Tz‐tk‐PEG (laser irradiation); and (4) TCO‐DOX@ZIF‐8 (pH 5.5) + ICG@Tz‐tk‐PEG (laser irradiation). As shown in Figure 2g, no TCO‐DOX or free DOX was detected in Group 1 and Group 3, indicating that the prodrug remained inactive under normal physiological conditions, regardless of NIR light exposure. In Group 2, a peak corresponding to the prodrug TCO‐DOX appeared, confirming the low pH‐induced release behavior, which was consistent with the earlier finding (Figure 2b). Notably, Group 4 exhibited minimal residual TCO‐DOX, in striking contrast to the appearance of a significant peak corresponding to DOX, demonstrating successful prodrug activation via the spontaneous IEDDA reaction between the liberated Tz and released TCO‐DOX. To demonstrate the ROS dependency, a control experiment was performed using a non‐cleavable micelle (ICG@PEG‐Tz without the TK linker, Figure S27 and S28, Supporting Information). In contrast to the successful activation in Group 4, the mixture of TCO‐DOX@ZIF‐8 and this control micelle at pH 5.5 with laser irradiation showed no change in the TCO‐DOX peak and no generation of free DOX. These results collectively confirm that effective activation of the prodrug based on our SC‐IEDDA strategy requires the synergistic action of both acidic pH and NIR laser irradiation.

### 2.3 Intracellular Prodrug Activation

The controllable behaviors of the two nanoplatforms in vitro prompted us to explore their intracellular performance, where 4T1 tumor cells representing a common malignancy of the breast were chosen as the model for demonstration. CCK‐8 viability assays showed that free TCO‐DOX exhibited significantly reduced cytotoxicity compared to native DOX (a half‐maximal inhibition concentration [IC_50_] = 1.26 μg mL^−1^), with an IC_50_ of 19.34 μg mL^−1^ (**Figure** **3**a). Such a result demonstrates the effective shielding of DOX activity by the TCO masking group. As anticipated, TCO‐DOX@ZIF‐8 displayed excellent cyto‐biocompatibility, with both 4T1 breast tumor and HEK293T cells maintaining >90% viability even at a concentration up to 100 μg mL^−1^ (Figure S29, Supporting Information), suitable for therapeutic utilization. Moreover, the cellular uptake study demonstrated that TCO‐DOX@ZIF‐8 was efficiently internalized, as evidenced by strong red fluorescence in the cytoplasm of 4T1 cells after coincubation (Figure S30, Supporting Information). Further subcellular localization validation revealed that the auto‐red fluorescence emitted from the DOX moiety in TCO‐DOX@ZIF‐8 was highly colocalized with the green fluorescence of the Lyso‐Tracker probe after 2 h of incubation (Figure 3b), indicating the nanoplatform's degradation in consideration of the acid environment of lysosomes (pH 4.5–5.5). Notably, after 8 h of incubation, the red fluorescence was diffused into the cytoplasm, suggesting successful endo‐lysosomal escape of TCO‐DOX, possibly due to the proton sponge effect of the imidazole ligands of ZIF‐8,^[^ ^40^ ^]^ which is beneficial for the diffusion of the DOX moiety into the cell nucleus to exert toxicity.

Figure 3
Intracellular controllable prodrug activation mediated by the SC‐IEDDA strategy. a) Cytotoxicity comparison of TCO‐DOX versus DOX toward 4T1 cells. b) Intracellular location profiling of TCO‐DOX@ZIF‐8 after incubation with 4T1 cells and corresponding colocalization analysis on the *X*‐axis fluorescence of Lyso‐Tracker and the DOX moiety for the selected cells. Scale bar = 75 μm. c) Fluorescence images of 4T1 cells incubated with Nile Red‐labeled ICG@Tz‐tk‐PEG before and after 808 nm laser irradiation (0.8 W cm^−2^, 2 min). d) Mean fluorescence intensity of 4T1 cells after incubation with Nile Red‐labeled ICG@Tz‐tk‐PEG before and after 808 nm laser irradiation, as determined by ImageJ software (*n* = 5). e) HR‐MS analysis of lysates obtained from 4T1 cells treated with ICG@Tz‐tk‐PEG under laser irradiation (0.8 W cm^−2^, 2 min). f) Concentration‐dependent cytotoxicity elicited by different conditions (denoted as Group 1: PBS; Group 2: ICG@Tz‐tk‐PEG + laser irradiation; Group 3: TCO‐DOX@ZIF‐8 + ICG@Tz‐tk‐PEG; Group 4: TCO‐DOX@ZIF‐8 + ICG@Tz‐tk‐PEG + laser irradiation) (*n* = 3 independent experiments). g) The subcellular distribution of the DOX moiety within 4T1 cells in Groups 1, 3, and 4 (left). Scale bar = 50 μm; Corresponding colocalization analysis on the *X*‐axis fluorescence of Hoechst and the DOX moiety for the selected cells (middle); HR‐MS analysis of cell lysates collected from Groups 1, 3, and 4 (right). h) Live/dead staining (Calcein‐AM/PI) of 4T1 cells treated as in Figure 3f. Scale bar = 275 μm. Data points were presented as mean ± s.d. Statistical analysis was calculated via unpaired *t* test and one‐way ANOVA with a Tukey post hoc test. **P < 0.01 and ****P < 0.0001 represent significant differences.

**Figure:**
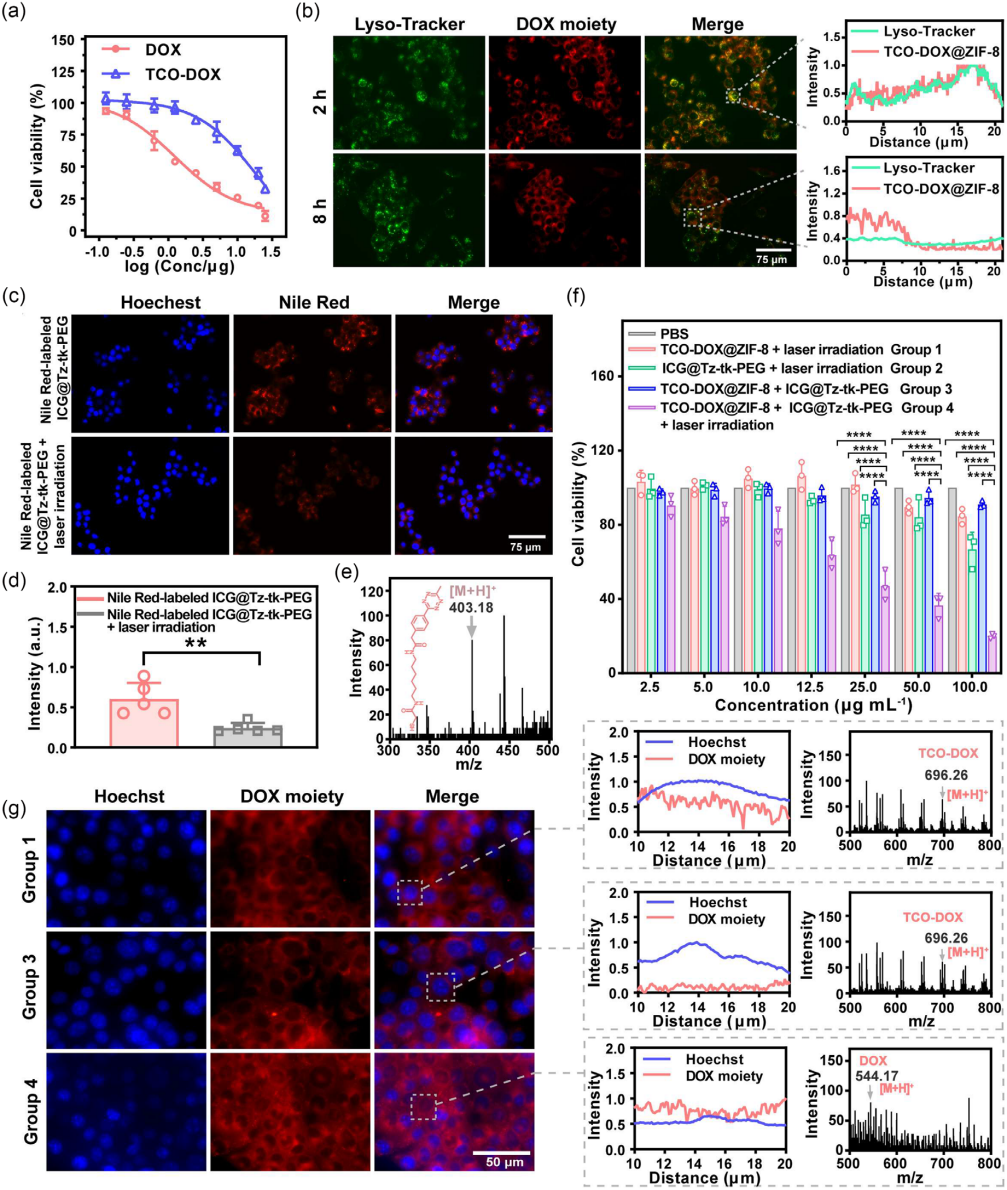

**Figure:**
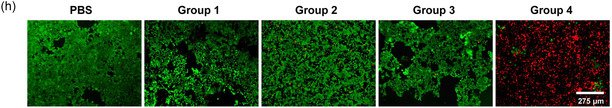

Parallel experiments revealed that ICG@Tz‐tk‐PEG could also be efficiently internalized by 4T1 cells (Figure S26, Supporting Information) and exhibited satisfactory cyto‐biocompatibility, with cells maintaining >90% viability even at a concentration up to 200 μg mL^−1^ (Figure S31, Supporting Information). As such, the intracellular NIR light‐responsiveness of ICG@Tz‐tk‐PEG was subsequently assessed. Fluorescence imaging profiling based on 2′,7′‐dichlorofluorescin diacetate (DCFH‐DA), the ROS indicator,^[^ ^57^ ^]^ revealed a concentration‐dependent increase of ROS production following 808 nm laser irradiation (0.8 W cm^−2^, 2 min) in ICG@Tz‐tk‐PEG‐treated cells (Figure S33, Supporting Information). Encouraged by this finding and to directly evaluate the NIR laser irradiation‐induced micellar disassembly within cells, Nile Red, a hydrophobic fluorescence probe exhibiting drastic fluorescence quenching upon micellar disintegration,^[^ ^50^ ^]^ was loaded into ICG@Tz‐tk‐PEG nanomicelles and incubated with 4T1 cells. As shown in Figure 3c,d, in the absence of NIR light exposure, intracellular Nile Red fluorescence remained stable, indicating intact micellar structures. In contrast, laser irradiation triggered a marked decrease in fluorescence intensity, suggesting the TK cleavage and micellar disassembly induced by the generation of intracellular ROS. Correspondingly, HR‐MS analysis of the lysates of ICG@Tz‐tk‐PEG‐treated 4T1 cells under NIR laser irradiation confirmed the successful liberation of the Tz activator (Figure 3e). Finally, we evaluated the cytotoxicity resulting from the controllable prodrug activation process. As shown in Figure 3f, treatment with either TCO‐DOX@ZIF‐8 (TCO‐DOX@ZIF‐8 + laser irradiation) or ICG@Tz‐tk‐PEG (ICG@Tz‐tk‐PEG + laser irradiation) alone under NIR laser irradiation caused only marginal cytotoxicity. Similarly, cotreatment with both nanoplatforms without irradiation (TCO‐DOX@ZIF‐8 + ICG@Tz‐tk‐PEG) showed minimal cytotoxicity, even at elevated concentrations, confirming the effective shielding of the interaction between the Tz activator and prodrug TCO‐DOX. Notably, upon 808 nm laser irradiation (TCO‐DOX@ZIF‐8 + ICG@Tz‐tk‐PEG + laser irradiation), cell viability significantly decreased in a concentration‐dependent manner, indicating successful NIR light irradiation‐induced liberation of Tz and subsequent uncaging of TCO‐DOX via the spontaneous IEDDA reaction. Subcellular localization studies supported this mechanism (Figure 3g). Specifically, fluorescence of the DOX moiety was predominantly located in the cytoplasm after incubation of TCO‐DOX@ZIF‐8 alone or coincubation of both nanoplatforms with 4T1 cells. On the contrary, NIR light‐irradiated cells cotreated with TCO‐DOX@ZIF‐8 and ICG@Tz‐tk‐PEG exhibited prominent nuclear accumulation of DOX, consistent with its known mechanism of action via nuclear DNA intercalation.^[^ ^58^ ^]^ HR‐MS analysis of 4T1 cell lysates from the TCO‐DOX@ZIF‐8 + ICG@Tz‐tk‐PEG + laser irradiation group directly confirmed the successful intracellular conversion of TCO‐DOX to DOX, whereas only TCO‐DOX was detected in the TCO‐DOX@ZIF‐8 + laser irradiation or the TCO‐DOX@ZIF‐8 + ICG@Tz‐tk‐PEG group (Figure 3g). To further corroborate the controllable tumor cell‐killing ability mediated by our SC‐IEDDA strategy, a live/dead staining assay was performed. In the TCO‐DOX@ZIF‐8 + ICG@Tz‐tk‐PEG + laser irradiation group, strong red fluorescence emitted from propidium iodide (PI)‐stained dead cells was observed (Figure 3h). In striking contrast, other groups exhibited green fluorescence of calcein‐acetoxymethyl ester (AM), reflecting the high cell viability. These findings substantiate the switchable cytotoxicity mediated by our SC‐IEDDA strategy, crucial for the precise cancer therapeutic application.

### 2.4 Biosafety and Biodistribution In Vivo

**Figure 4:**
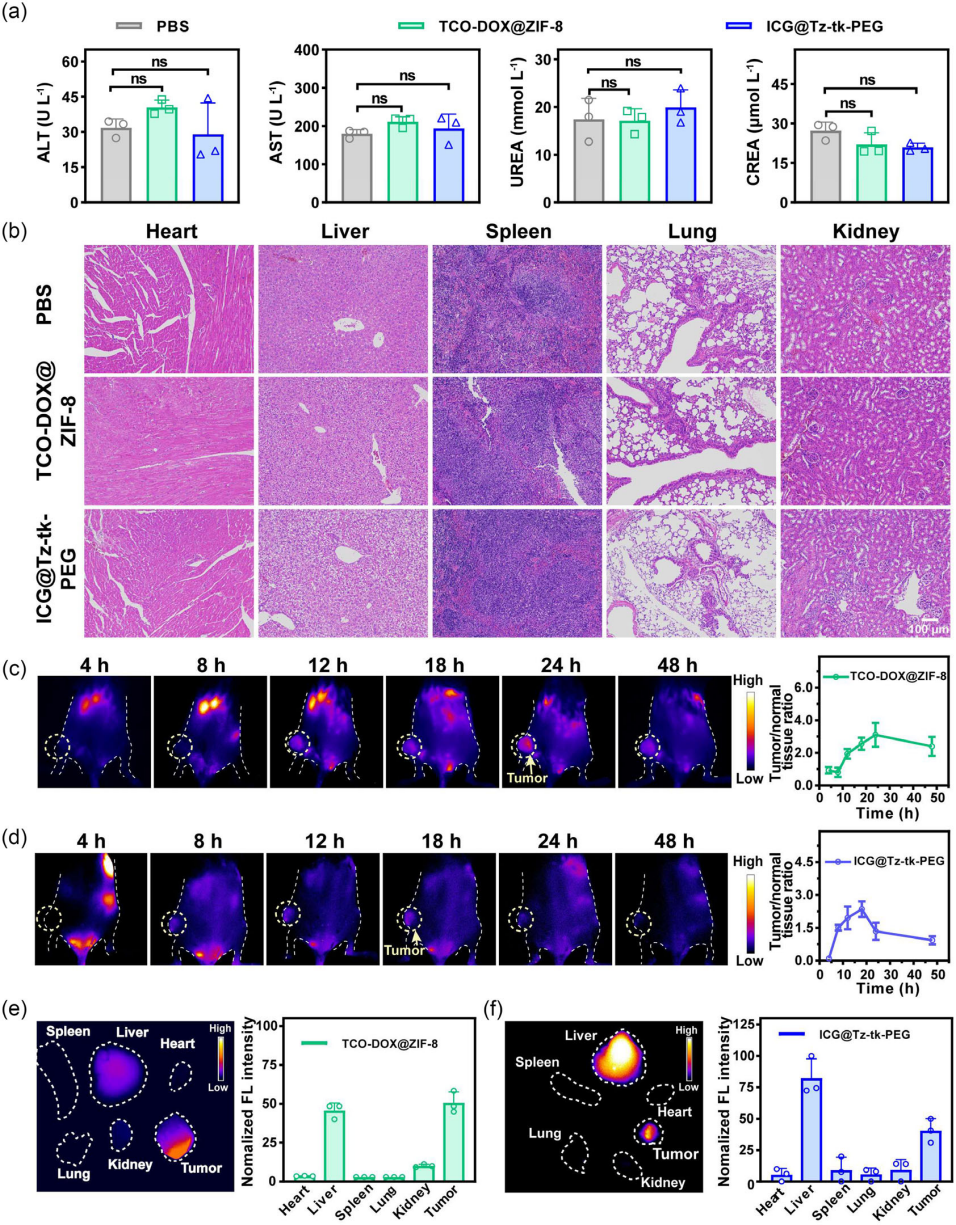
In vivo evaluation of the biosafety and distribution profiles of TCO‐DOX@ZIF‐8 and ICG@Tz‐tk‐PEG. a) The levels of hepatic and renal function markers in normal mice administered PBS, TCO‐DOX@ZIF‐8, or ICG@Tz‐tk‐PEG. b) H&E‐stained sections of major organs (i.e., heart, liver, spleen, lung, kidney) harvested from normal mice after intravenous injection of PBS, TCO‐DOX@ZIF‐8, or ICG@Tz‐tk‐PEG. Scale bar = 100 μm. c) In vivo time‐dependent NIR‐II fluorescence imaging of 4T1 tumor‐bearing mice postinjection of TCO‐DOX@ZIF‐8 and the corresponding tumor‐to‐normal tissue accumulation ratio over time. d) In vivo time‐dependent fluorescence imaging of 4T1 tumor‐bearing mice postinjection of ICG@Tz‐tk‐PEG and the corresponding tumor‐to‐normal tissue accumulation ratio over time. e) Ex vivo fluorescence imaging of major organs and tumors collected from 4T1 tumor‐bearing mice at 24 h postinjection of TCO‐DOX@ZIF‐8 and the corresponding semiquantitative fluorescence analysis. f) Ex vivo fluorescence imaging of major organs and tumors collected from 4T1 tumor‐bearing mice at 18 h postinjection of ICG@Tz‐tk‐PEG and the corresponding semi‐quantitative fluorescence analysis (*n*  =  3 mice per group). Data points were presented as mean ± s.d. Statistical analysis was calculated via one‐way ANOVA with a Tukey post hoc test. “ns” represents no significant difference.

Encouraged by the favorable in vitro outcomes, we proceeded to conduct the in vivo studies. We first systematically evaluated the biosafety profiles of TCO‐DOX@ZIF‐8 and ICG@Tz‐tk‐PEG. Normal mice that received intravenous administration of either TCO‐DOX@ZIF‐8 (10 mg kg^−1^) or ICG@Tz‐tk‐PEG (20 mg kg^−1^) exhibited normal growth curves, with no significant deviation from PBS‐treated normal mice throughout the study period (Figure S34, Supporting Information). Serum biochemical analyses revealed no statistically significant changes in liver function markers (i.e., alanine aminotransferase [ALT], aspartate transaminase [AST], and albumin [ALB]), kidney function markers (urea [UR], creatinine [CR], and uric acid [UA]), lipids, glucose, and cardiac enzymes profiles among all the mice, indicating negligible systemic toxicity elicited by TCO‐DOX@ZIF‐8 or ICG@Tz‐tk‐PEG (**Figure** **4**a and Figure S35–S38, Supporting Information). Histological examination of major organs (i.e., heart, liver, spleen, lung, kidney) via hematoxylin and eosin (H&E) staining showed no signs of inflammation, necrosis, or other pathological abnormalities (Figure 4b). These results strongly confirm the excellent biocompatibility of both developed nanoplatforms, validating their suitability for subsequent therapeutic investigations and potential clinical translations.

To intuitively evaluate the biodistribution profiles of the two nanoplatforms after intravenous administration into mice bearing 4T1 tumors, we performed fluorescence imaging in the second near‐infrared (NIR‐II, 1000‐1700 nm) window due to its high spatiotemporal resolution.^[^ ^51^ ^]^ The IR‐820 dye was coencapsulated in TCO‐DOX@ZIF‐8 to facilitate real‐time in vivo tracking. As shown in Figure 4c, the IR‐820 fluorescence signal in tumor tissues increased progressively over time, reaching a maximum at 24 h post‐injection and sustaining high intensity thereafter. This behavior is indicative of passive accumulation at the tumor site via the EPR effect. Similarly, as revealed by fluorescence of ICG, ICG@Tz‐tk‐PEG demonstrated gradual tumor accumulation, peaking at ≈18 h postinjection (Figure 4d), likely facilitated by its nanoscale size as well as prolonged circulation time afforded by PEGylation.^[^ ^59^ ^]^ Semiquantitative analysis of excised organs and tumors further confirmed significant tumor accumulation of both nanoplatforms (Figure 4e,f), alongside certain uptake by the livers, a common feature for nanoparticles due to the clearance by the reticuloendothelial system.^[^ ^60^ ^]^ Collectively, the distinct temporal profiles of tumor accumulation for each nanoplatform support the implementation of an interval between their administrations. This approach ensures optimal spatiotemporal coordination to activate prodrug for controlled anti‐cancer therapy.

### 2.5 Precise Chemotherapy for the Effective Antitumor Process

Finally, to investigate the superiority of our SC‐IEDDA strategy in achieving precise and effective antitumor efficacy, 4T1 tumor‐bearing mice were randomly divided into seven groups and separately underwent treatments as follows: 1) PBS, 2) TCO‐DOX@ZIF‐8, 3) ICG@Tz‐tk‐PEG + laser irradiation, 4) TCO‐DOX@ZIF‐8 + ICG@Tz‐tk‐PEG, 5) TCO‐DOX@ZIF‐8 + ICG@Tz‐tk‐PEG + laser irradiation, 6) free DOX, and 7) DOX@ZIF‐8. The resulting DOX‐equivalent payload of TCO‐DOX@ZIF‐8 (10 mg kg^−1^) was administered to Groups 2, 4, and 5, matching the free DOX dose (2 mg kg^−1^) applied in Group 6 and the DOX@ZIF‐8 (12.2 mg kg^−1^, DLC = 16.4%) in Group 7. Particularly, based on the prior in vivo biodistribution (Figure 4c,d) and in vitro cytotoxicity data (Figure 3f), Groups 4 and 5 received sequential intravenous administration of TCO‐DOX@ZIF‐8 and ICG@Tz‐tk‐PEG at a 6 h interval, and laser irradiation (808 nm, 0.8 W cm^−2^, 2 min) was applied at 18 h post‐ICG@Tz‐tk‐PEG injection and 24 h post‐TCO‐DOX@ZIF‐8 injection in Groups 3 and 5, respectively. Treatments were conducted every 3 days over a 2‐week period according to the schedule shown in **Figure** **5**a. As illustrated in Figure 5b,c, neither TCO‐DOX@ZIF‐8 alone nor its combination with ICG@Tz‐tk‐PEG (without laser irradiation) suppressed tumor growth, indicating minimal therapeutic effect in the absence of prodrug activation. Similarly, the ICG@Tz‐tk‐PEG + laser irradiation group exhibited limited tumor inhibition. In stark contrast, the fully activated system—TCO‐DOX@ZIF‐8 + ICG@Tz‐tk‐PEG + laser irradiation—resulted in substantial tumor suppression, even surpassing the therapeutic efficacy of free DOX and being comparable to that of DOX@ZIF‐8. This enhanced performance likely arises from the preferential enrichment of the DOX moiety in the tumors via the nanoplatform delivery. Moreover, mice treated with TCO‐DOX@ZIF‐8 + ICG@Tz‐tk‐PEG + laser irradiation maintained a stable body weight (Figure 5d). In contrast, mice administered free DOX suffered significant body weight loss. A decrease in body weight was also observed in the DOX@ZIF‐8 group due to the premature and nonspecific leakage of the active drug. This highlights the safety advantage conferred by spatiotemporal control of the prodrug activation.

Figure 5
SC‐IEDDA strategy‐mediated prodrug activation in the tumor for the anticancer process. a) Schematic illustration of the therapeutic procedure. The 4T1 tumor‐bearing mouse model was established by subcutaneously injecting 4T1 cells into the right flank of the mice. Seven days later, the mice were randomly divided into seven groups (*n* = 5 mice per group) and underwent the following treatments every 3 days over a 2‐week period: Group 1, PBS; Group 2, TCO‐DOX@ZIF‐8; Group 3, ICG@Tz‐tk‐PEG + laser irradiation; Group 4, TCO‐DOX@ZIF‐8 + ICG@Tz‐tk‐PEG; Group 5, TCO‐DOX@ZIF‐8 + ICG@Tz‐tk‐PEG + laser irradiation; Group 6, free DOX; and Group 7, DOX@ZIF‐8. Groups 4 and 5 received sequential intravenous administration of TCO‐DOX@ZIF‐8 and ICG@Tz‐tk‐PEG at a 6 h interval. Laser irradiation (808 nm, 0.8 W cm^−2^, 2 min) was applied at 18 h post‐ICG@Tz‐tk‐PEG and 24 h post‐TCO‐DOX@ZIF‐8 injection in Groups 3 and 5, respectively. Tumor volume was monitored every 2 days. b) Changes in average tumor sizes across different groups (*n* =  5 mice per group). c) Representative photos of the excised tumors on day 15 after the various treatments (*n* =  5 mice per group). d) Variation in the body weight of 4T1 tumor‐bearing mice over time across different groups (n  =  5 mice per group). e) H&E, TUNEL, and Ki‐67 staining of tumors harvested from different treatment groups. Scale bar = 100 μm. f) H&E staining of major organs (i.e., heart, liver, spleen, lung, and kidney) harvested from Groups 1, 5, 6, and 7. The free DOX group showed disorganized myocardial fibers and cytoplasmic vacuolization, monocyte/macrophages in splenic tissues, and dilated, congested renal tubules (black arrow). g) DOX concentrations in major organs of mice from Group 5 at 6 h and 12 h post laser irradiation (*n* =  3 mice per group). h) DOX concentrations in major organs of mice from Group 6 at 6 h and 12 h postinjection of DOX (n = 3 mice per group). Data points were presented as mean  ±  s.d. Statistical analysis was calculated via one‐way ANOVA with a Tukey post‐hoc test. ***P < 0.001 and ****P < 0.0001 represent a significant difference, and “ns” represents no significant difference.

**Figure:**
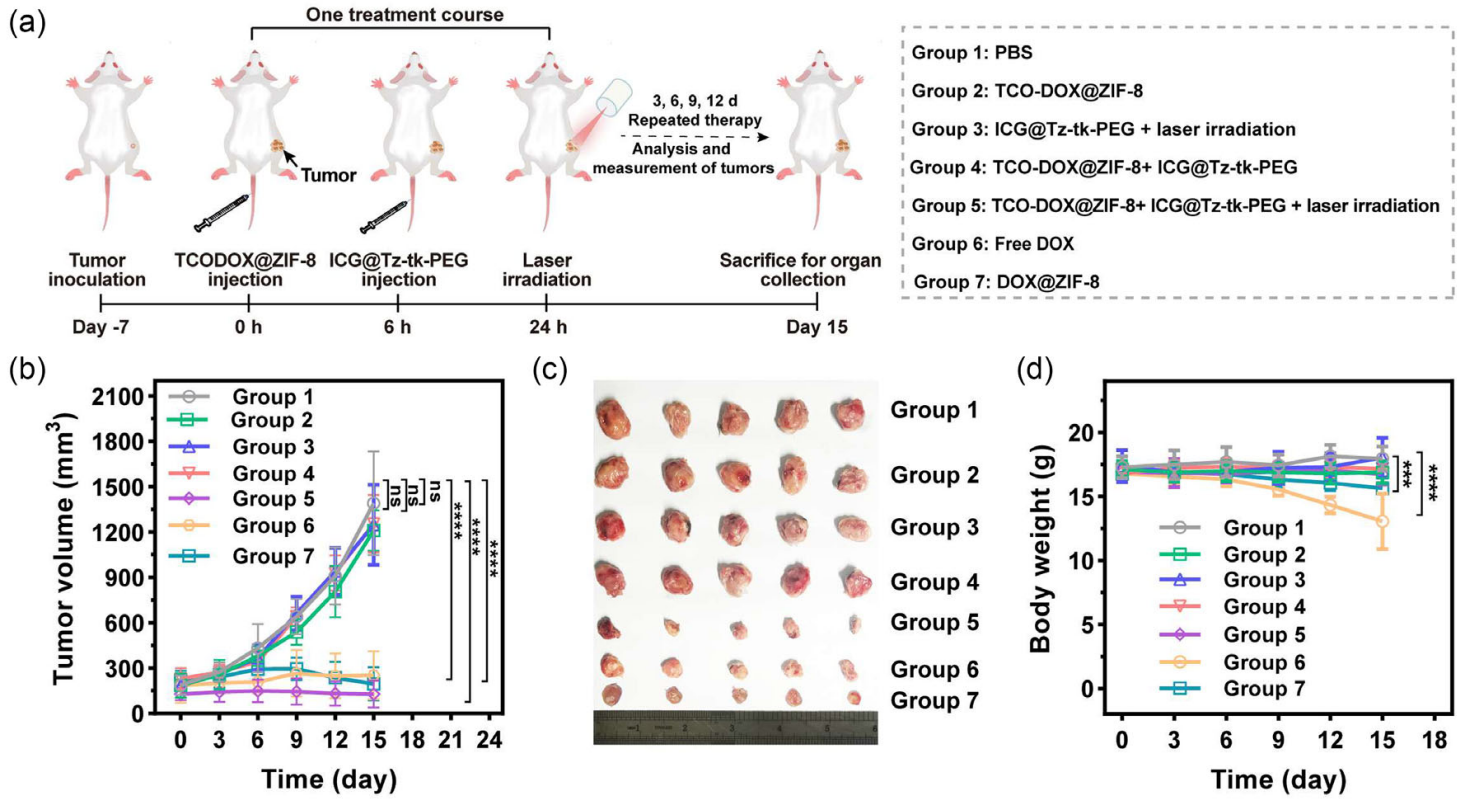

**Figure:**
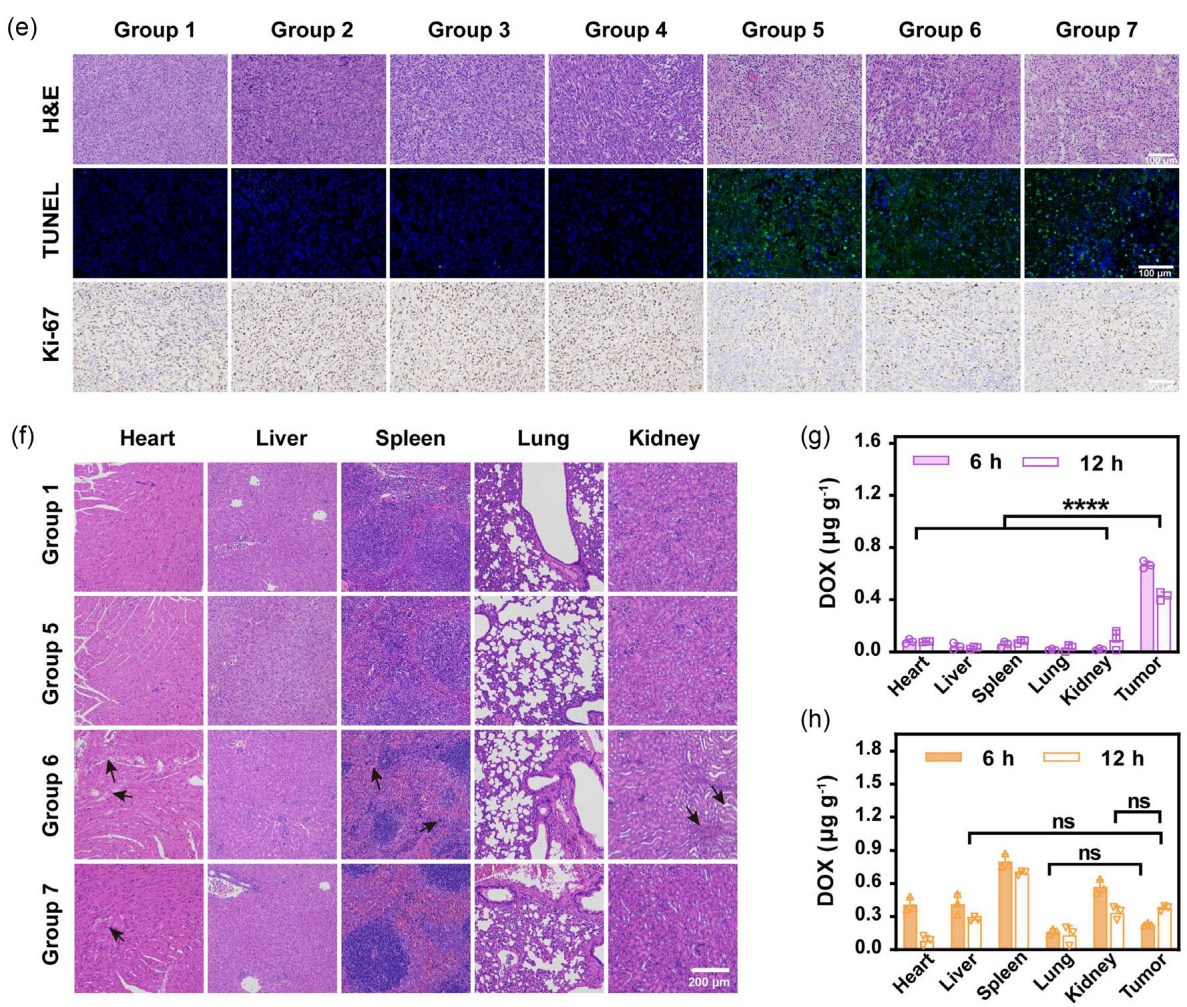

Histological analyses further corroborated the therapeutic outcomes. Compared to other groups, the H&E staining of tumor sections harvested from Group 5 showed the most obvious cell contraction and nucleus deletion (Figure 5e). TUNEL assay and Ki‐67 immunohistochemical assay revealed significantly more extensive apoptosis and reduced proliferation in Groups 5, 6, and 7, consistent with the tumor growth inhibition results. Despite the comparable antitumor efficacy at the tumor site, distinct differences in systemic toxicity were observed among these groups (Figure 5f). Free DOX treatment caused severe cardiotoxicity, as evidenced by disorganized myocardial fibers and cytoplasmic vacuolization. Histological abnormalities were also observed in the spleens and kidneys by free DOX treatment, including aberrant clusters of monocyte/macrophages in splenic tissues and dilated congested renal tubules. Although the DOX@ZIF‐8 group demonstrated improved biosafety over free DOX, it evidently presented a residual cardiotoxic effect, as indicated by slight disorganization of myocardial fibers (Figure 5f). By contrast, no significant histological damage was detected in the major organs of mice in Group 5. The superior safety profile of Group 5 was further corroborated by quantitative high‐performance liquid chromatography (HPLC) analysis of tissue lysates, which confirmed tumor‐specific DOX generation with minimal off‐target accumulation in major organs (Figure 5g). This tumor selectivity is attributed to the dual‐trigger activation mechanism, reliant on both acidic pH and NIR light irradiation. In contrast, free DOX is distributed broadly throughout the body, with high off‐target deposition (Figure 5h). Collectively, these findings demonstrate that our SC‐IEDDA strategy enables potent and tumor‐specific chemotherapy via spatiotemporally controlled bioorthogonal prodrug activation, while simultaneously reducing systemic toxicity—representing a key step toward safe and effective clinical translation.

## 3 Conclusion

In summary, we have developed a spatiotemporally controlled bioorthogonal prodrug activation (SC‐IEDDA) strategy for precise chemotherapy. Following passive accumulation of the pH‐sensitive prodrug‐loaded nanoplatform (TCO‐DOX@ZIF‐8) and NIR light‐responsive nanomicelle (ICG@Tz‐tk‐PEG) bearing the prodrug activator in the tumors via the EPR effect, the acidic environment induced degradation of TCO‐DOX@ZIF‐8 to release the prodrug TCO‐DOX, while NIR laser irradiation triggered the disassembly of ICG@Tz‐tk‐PEG for the prodrug activator Tz liberation. This dual‐stimuli design enabled in situ generation of DOX in the tumors via the spontaneous IEDDA reaction between TCO‐DOX and Tz, confining therapeutic activation to the tumor site. In vivo studies confirmed effective tumor growth inhibition with minimal systemic toxicity.

Despite these promising results, we acknowledge the complexities of this approach and the translational challenges. The current strategy employs a dual‐injection regimen, potentially complicating clinical protocols and patient compliance. Further simplification of the administration process is necessary to enhance practical applicability. In addition, while the EPR effect facilitated tumor accumulation in the present model, its reliability varies across tumor types and patients, requiring assessment in more physiologically relevant models, such as orthotopic or spontaneous metastasis models, to better recapitulate the heterogeneity and immunosuppressive nature of advanced tumor microenvironments. Rigorous assessment of penetration depth, dosing control, and immune modulation in such settings will be crucial for clinical adaptation. Overall, while this stimulus‐gated bioorthogonal strategy offers a versatile platform for spatially controlled therapy, further research is essential to address these translational hurdles and broaden its application to other diseases requiring spatiotemporal control of therapeutic agents.

## 4 Experimental Section

4.1

4.1.1

##### Preparation of TCO‐DOX@ZIF‐8

Initially, 2‐MIM (330 mg) and TCO‐DOX (10 mg) were dissolved in methanol (10 mL). Then, an aqueous solution of Zn(NO_3_)_2_·6H_2_O, prepared by dissolving 150 mg in 5 mL deionized water, was added dropwise to the methanol solution under continuous magnetic stirring. After a 10 min reaction at room temperature, the resulting TCO‐DOX@ZIF‐8 nanoparticles were collected by centrifugation (12000 rpm, 10 min) and washed sequentially with methanol and deionized water (three times each). The DLC was quantified by completely dissolving the ZIF‐8 carrier and quantifying the released TCO‐DOX. Specifically, 2.0 mg of TCO‐DOX@ZIF‐8 nanoparticles was dissolved in a pH 5.0 buffer (containing 1% Tween‐80) and incubated at 37 °C with constant shaking for 12 h until the solution became clear, indicating complete dissolution of the framework. The concentration of released TCO‐DOX in the supernatant was then measured using UV‐Vis spectroscopy at 480 nm, with reference to a pre‐established standard calibration curve. The DLC was calculated according to the following formula

DLC (%) = (mass of loaded drug/total mass of drug‐loaded nanoparticles) × 100%

##### Preparation of ICG@Tz‐tk‐PEG

Tz‐tk‐PEG (2.5 mg) and ICG (0.25 mg) were codissolved in 100 μL DMF, followed by drying under a gentle nitrogen stream at room temperature. Subsequently, 2 mL deionized water was added to the dried mixture and subjected to bath sonication (40 kHz, 100 W) for 30 min at 25 °C. The resulting solution was dialyzed against deionized water for 24 h to remove unassembled molecules, yielding purified ICG@Tz‐tk‐PEG. The concentration of ICG in dialysate after dialysis was determined by measuring the absorbance at 780 nm using UV‐Vis spectroscopy. Dry micelles were obtained by freeze‐drying. The DLC was calculated using the following equation

DLC (%) = (mass of loaded component)/(mass of formulation) × 100%

##### pH‐Responsive Release of TCO‐DOX

The pH‐responsive release of TCO‐DOX was systematically investigated under simulated conditions. The TCO‐DOX@ZIF‐8 nanoplatforms were separately suspended in PBS adjusted to normal physiological (pH 7.4), tumor microenvironment (pH 6.5), and lysosomal (pH 5.5) conditions, supplemented with 1.0% v/v Tween 80. At predetermined intervals, the released drug fraction was isolated via centrifugal separation (12 000 rpm, 10 min). UV‐Vis spectrophotometric analysis (*λ* = 480 nm) was employed to quantify drug concentrations using a pre‐validated standard curve.

##### NIR Light‐Sensitivity of ICG@Tz‐tk‐PEG

ICG@Tz‐tk‐PEG nanomicelles (100 μg mL^−1^) were irradiated with an 808 nm laser (0.8 W cm^−2^) for 2 min. Subsequently, the hydrodynamic diameter distribution of postirradiation nanomicelles was determined using DLS analysis. For morphological analysis, TEM samples were prepared from irradiated solutions to assess structural variation. Finally, laser irradiation‐triggered degradation products were characterized by HR‐MS.

##### Evaluation of Prodrug Activation in Vials

TCO‐DOX@ZIF‐8 (100 μg mL^−1^) was added into PBS (pH 7.4 or 5.5). In parallel, ICG@Tz‐tk‐PEG (200 μg mL^−1^) was added into PBS, followed by laser irradiation at 808 nm for 2 min with the power of 0.8 W cm^−2^ or not. The two solutions were separately incubated at 37 °C for 24 h. After the incubation, the two solutions were mixed at equal volume and then incubated for another 6 h. Finally, the prodrug activation was profiled by HPLC.

##### Cell Culture

All cell lines were obtained from Wuhan Pricella Biotechnology Co., Ltd. (Wuhan, China). 4T1 cells were incubated in Dulbecco's modified Eagle medium (DMEM) supplemented with 10% FBS and 1% penicillin/streptomycin. HEK293T cells were cultured in RPMI 1640 medium supplemented with 10% FBS and 1% penicillin and streptomycin. All cells were cultured at 37 °C under 5% CO_2_. The culture medium was changed at ≈1 to 2 day intervals.

##### Cellular Uptake

The uptake of TCO‐DOX@ZIF‐8 or ICG@Tz‐tk‐PEG by 4T1 cells was investigated using fluorescence imaging. 4T1 cells (5 × 10^3^) were seeded onto a 96‐well plate and cultured for 24 h at 37 °C in the incubator. Then, the original medium was replaced with fresh culture medium containing TCO‐DOX@ZIF‐8 or ICG@Tz‐tk‐PEG and further incubated for 2, 4, and 6 h, respectively. Subsequently, the 4T1 cells were washed three times with PBS at room temperature and stained with Hoechst 33342 for 15 min. Finally, the cells were imaged using an inverted epifluorescence microscope (Thermo/EVOS M7000, Thermo Fisher Scientific, USA).

##### Subcellular Localization

Cells were plated and incubated for 12 h. The medium was then aspirated and replaced with fresh medium containing TCO‐DOX@ZIF‐8. Following incubation periods of 2 or 8 h, the medium was removed. Cells were subsequently incubated with medium containing the Lyso‐Tracker probe for 30 min to label the lysosome. Finally, the cells were washed thoroughly with PBS (three times) and subjected to an inverted epifluorescence microscope for imaging (Thermo/EVOS M7000, Thermo Fisher Scientific, USA).

##### Cytotoxicity Assay

4T1 cells were seeded in 96‐well plates at a density of 5 × 10^3^ cells per well. After incubation at 37 °C for 12 h, the cells were initially treated with TCO‐DOX@ZIF‐8 nanoplatforms for 4 h. Following this incubation, the medium was replaced with fresh medium containing ICG@Tz‐tk‐PEG nanomicelles (the optimal mass ratio of TCO‐DOX@ZIF‐8 to ICG@Tz‐tk‐PEG was 1:2), and the cells were then incubated for an additional 4 h. Afterward, the cells were washed three times with PBS and fresh medium was added, with subsequent irradiation with an 808 nm laser (0.8 W cm^−2^) for 2 min. After irradiation, the cells were returned to the incubator and cultured for 12 h. Then, the medium was aspirated, the cells were washed three times with PBS, and fresh medium supplemented with 10% CCK‐8 detection solution was added for a 2 h incubation. Finally, cell viability was determined from absorbance values measured at 450 nm using Thermo/Varioskan LUX multimode microplate reader (Thermo Fisher Scientific, USA).

##### Live/Dead Cell Staining

Live/dead cell staining was performed on experimental groups that underwent identical therapeutic treatments described for the cytotoxicity assay. After different treatments, the culture medium was aspirated, and cells were costained with Calcein‐AM (indicating viable cells) and PI (indicating dead cells) in serum‐free medium for 30 min at 37 °C in the dark. Finally, cells were washed three times with PBS and observed using an inverted epifluorescence microscope (Thermo/EVOS M7000, Thermo Fisher Scientific, USA).

##### Animal Experiments

Female BALB/c mice (4 weeks) were purchased from Yingke Rui Biotech (Beijing, China) and housed under standard conditions at the Animal Center of Biomedical Analysis in Nanchang University (Nanchang, China). Mice were randomly assigned to different groups to carry out experimental investigations. Investigators were blinded to group allocations during data collection and analysis. All animal procedures were approved by the Institutional Animal Care and Use Committee of Nanchang University (Approval number: SYXK (Gan) 2021‐0004).

##### In Vivo Biocompatibility Evaluation

To evaluate the biocompatibility, TCO‐DOX@ZIF‐8 (10 mg kg^−1^, equivalent dose of DOX: 2 mg kg^−1^) and ICG@Tz‐tk‐PEG (20 mg kg^−1^) were injected into normal mice via the tail vein, respectively. At 15 days postinjection, mice were sacrificed, and major organs were collected for H&E staining. Mouse blood was also collected for routine analysis of liver function, kidney function, and other biochemical indicators.

##### In Vivo Biodistribution of TCO‐DOX@ZIF‐8 and ICG@Tz‐tk‐PEG after Intravenous Injection

4T1 tumor‐bearing mice were randomly divided into two groups (*n *= 3 per group) and separately received an intravenous injection of IR‐820/TCO‐DOX@ZIF‐8 (10 mg kg^−1^) and ICG@Tz‐tk‐PEG (20 mg kg^−1^). At 4, 8, 12, 16, 24, and 48 h postinjection, mice were subjected to in vivo real‐time imaging using a NIR‐II fluorescence imaging system (DeepVision imaging system, Nirmidas, Inc., USA). Tumors and other major organs (i.e., heart, liver, spleen, lung, and kidney) were harvested at designated time points for ex vivo fluorescence imaging.

##### In Vivo Antitumor Efficacy

BALB/c mice were subcutaneously inoculated with 5 × 10^5^ 4T1 cells at the right thigh. When tumor volumes reached ≈80 mm^3^, mice were randomly divided into seven groups (*n* = 5): Group 1: PBS; Group 2: TCO‐DOX@ZIF‐8; Group 3: ICG@Tz‐tk‐PEG + laser irradiation; Group 4: TCO‐DOX@ZIF‐8 + ICG@Tz‐tk‐PEG; Group 5: TCO‐DOX@ZIF‐8 + ICG@Tz‐tk‐PEG + laser irradiation; Group 6: free DOX, and Group 7: DOX@ZIF‐8. All materials were administered intravenously via tail vein. Groups 2, 4, and 5 were administered 10 mg kg^−1^ TCO‐DOX@ZIF‐8 nanoparticles. The resulting DOX‐equivalent payload (2 mg kg^−1^) matched the free DOX dose applied in Group 6. In addition, the mice in Groups 3, 4, and 5 were injected with ICG@Tz‐tk‐PEG at a dose of 20 mg kg^−1^. Groups 4 and 5 received sequential intravenous injections of TCO‐DOX@ZIF‐8 and ICG@Tz‐tk‐PEG at a 6 h interval, and laser irradiation (808 nm, 0.8 W cm^−2^, 2 min) was applied at 18 h post‐ICG@Tz‐tk‐PEG and 24 h post‐TCO‐DOX@ZIF‐8 injection in Groups 3 and 5, respectively. Tumor volumes and body weights were measured every 3 days, with tumor volume calculated as (width^2^ × length)/2.

##### Statistical Analysis

A minimum of three independent replicates were performed for each experiment, with the exact sample size (*n*) for each dataset provided in the figure legends. Quantitative data are presented as the mean ± standard deviation (mean ± SD), and all error bars represent the SD. Statistical analyses were conducted using GraphPad Prism 9.0 and Origin 2018. For comparisons between two independent groups, an unpaired two‐tailed Student's *t*‐test was used. Comparisons across three or more groups involving a single factor were performed using one‐way ANOVA. A *P* value < 0.05 was considered statistically significant, with specific levels denoted as follows: *P < 0.05, **P < 0.01, ***P < 0.001, and ****P < 0.0001.

## Supporting Information

Supporting Information is available from the Wiley Online Library or from the author.

## Conflict of Interest

The authors declare no conflict of interest.

## Supporting information

Supplementary Material

## Data Availability

Data available on request from the authors.
