# Supplementary material for: Spatiotemporally Controlled Bioorthogonal Prodrug Activation for Precise Chemotherapy
Source: Small Sci. 2025 Nov 21;6(1):e202500483. doi: 10.1002/smsc.202500483 (PMC12798786; doi:10.1002/smsc.202500483)
Supplement: Supplementary file 1 — Supplementary Material [file SMSC-6-e202500483-s001.pdf]

Spatiotemporally controlled bioorthogonal prodrug activation for precise chemotherapy

Xia Liu,[a, b] Xiao Liang,[c] Ziqi Fang,[c] Fan Liu,[c] Wenbin Zhong,[b] Yiqun Wan,[c] and Hao Wan*[b]

Chemicals. DOX, 2-methylimidazole (2-MIM, 98%), methoxy polyethylene glycol amine (MPEG2000-NH_2) (MW = 2000), and thioglycolic acid (TGA) were procured from Aladdin Industrial Corporation (Shanghai, China). (R, E)-Cyclooct-2-en-1-yl (4-nitrophenyl) carbonate was purchased from Sigma-Aldrich (St. Louis, MO, USA). Zinc nitrate hexahydrate (Zn $(NO_3)_2$) was acquired from Xi Long Scientific Co., Ltd. (Guangdong, China). N-BOC-1,6-diaminohexane, trifluoroacetic acid, 2-(4-cyanophenyl)acetic acid, nickel(II) trifluoromethanesulfonate $Ni(OTf)_2$, and ICG were purchased from Shanghai Bide Pharma tech Co., Ltd. o-(7-Azabenzotriazol-1-yl)-N,N,N',N'-tetramethyluronium hexafluorophosphate (HATU), N-hydroxy succinimide (NHS), N-(3-dimethylaminopropyl)-N'-ethylcarbodiimide hydrochloride (EDCI), 1-hydroxybenzotriazole (HOBT), trifluoroacetic acid (TFA), triethylamine (TEA), 4-(dimethylamino)pyridine (DMAP), and ethyldiisopropylamine (DIEA) were purchased from Macklin Biochemical Co., Ltd. (Shanghai, China). Anhydrous solvents, such as dichloromethane (DCM), acetonitrile (MeCN), and N, N-dimethylformamide (DMF) were purchased from Sigma-Aldrich. The solvents for purification, including DCM, petroleum ether (PE), methanol (MeOH), and ethyl acetate (EA) were of analytical grade. Triple-distilled water was used throughout the experiments.

Characterizations. ^1H and ^{13}C NMR spectra were measured with Bruker/AVANCE NEO 400 and AVANCE NEO 600 Spectrometer (Bruker AG, Germany). The HR-MS analysis was conducted on the UHPLC30A-5600+ system (AB SCIEX, USA). The absorption spectra were collected on Lambda1050+ (PerkinElmer, United Kingdom). TEM images were collected on JEM-F200 (JEOL, Japan). Size distribution of the nanoplatforms was determined through DLS analysis by using Malvern Instrument Zetasizer Pro at 25 ^oC (Malvern Panalytical Limited, United Kingdom). HPLC data were acquired from Agilent 1260 Infinity liquid chromatograph (Agilent Technologies, USA).

Synthesis of Compound TCO-DOX. (E)-Cyclooct-2-en-1-yl (4-nitrophenyl) carbonate (80 mg, 257.6 μmol) was dissolved in anhydrous DMF (10 mL) in a 25 mL single-necked round-bottom flask, followed by the addition of DIEA (2 mL) and DOX (140 mg, 257.6 μmol). The reaction mixture was shielded from light and stirred at 30 ^oC for 24 h. Upon completion, the reaction was quenched with deionized water (40 mL), and the mixture was extracted with DCM (3×15 mL). The combined organic layers were washed with deionized water (3×15 mL), then dried over anhydrous Na_2SO_4 and filtered. The solvent was removed under reduced pressure, and the crude product was purified by silicone column chromatography (DCM/MeOH = 40:1, v/v) to

afford compound TCO-DOX (90.2 mg, 53.3%). ^1H NMR (600 MHz, $CDCl_3$): δ 13.96 (s, 1H), 13.21 (s, 1H), 8.11 – 7.98 (m, 1H), 7.78 (t, J = 8.0 Hz, 1H), 7.38 (dd, J = 8.6, 3.7 Hz, 1H), 5.49 (d, J = 23.8 Hz, 2H), 5.42 – 5.23 (m, 2H), 5.12 (d, J = 8.4 Hz, 1H), 4.75 (d, J = 1.8 Hz, 2H), 4.53 (s, 1H), 4.27 – 4.03 (m, 4H), 3.87 (s, 1H), 3.68 (d, J = 2.6 Hz, 1H), 3.35 – 3.22 (m, 1H), 3.00 (d, J = 18.7 Hz, 2H), 2.58 – 2.30 (m, 2H), 2.19 (d, J = 11.2 Hz, 1H), 2.10 – 1.79 (m, 7H), 1.72 – 1.57 (m, 3H), 1.44 (s, 1H), 1.29 (dd, J = 6.5, 4.6 Hz, 4H), 0.76 (s, 1H). ^{13}C NMR (151 MHz, $CDCl_3$): δ 187.04, 186.70, 162.54, 161.13, 156.17, 155.68, 135.68, 131.77, 119.85, 118.54, 111.65, 111.46, 74.09, 69.71, 69.58, 65.48, 56.66, 36.38, 35.85, 34.06, 31.42, 29.65, 29.09, 24.06, 16.80. HR-MS (m/z): Calcd for $C_{36}H_{41}NO_{13}$. Exact Mass: 695.2578, found: 694.2579 $[M-H]^-$.

Synthesis of Compound 2,2'-(propane-2,2-diylbis[sulfanediyl]) diacetic acid (PSDA). A solution of TGA (1 g, 10.9 mmol) and TFA (100 µL) in acetone (320 mg, 5.4 mmol) was reacted at ambient temperature for 3 h. The reaction was quenched by adding deionized water (20 mL) to precipitate crystalline solids. Crude material was purified via three washes with anhydrous diethyl ether (20 mL × 3), yielding PSDA (2 g, 82.1%) as a white solid. ^1H NMR (400 MHz, $CDCl_3$): δ 3.53 (s, 1H), 1.63 (s, 1H).

Synthesis of Compound 1. A 250 mL three-necked round-bottom flask equipped with a magnetic stirrer was added with 2-(4-cyanophenyl) acetic acid (2 g, 12.4 mmol), anhydrous MeCN (6.4 mL), $Ni(OTf)_2$ (2.3 g, 6.1 mmol), and hydrazine hydrate (80% w/w, 27.3 mL). The mixture was purged via three vacuum-nitrogen cycles and stirred at 60 ^oC for 24 h under nitrogen protection. After cooling to room temperature, a solution of $NaNO_2$ (12.5 g in 72.8 mL H_2O) was added dropwise under ice-bath cooling. The pH was adjusted to 2-3 with 1 M HCl (ice bath, ≤5 ^oC), yielding a red solution. The mixture was extracted with ethyl acetate (3×100 mL), and the combined organic layers were washed with saturated brine (3×100 mL), dried over anhydrous Na_2SO_4 (10 g), filtered, and concentrated. The residue was purified by silica gel chromatography (DCM/MeOH, 95:5 v/v) to afford compound 1 (500 mg, 35%) as a purple solid. ^1H NMR (600 MHz, $CDCl_3$): δ 8.57 (d, J = 8.3 Hz, 2H), 7.52 (d, J = 8.4 Hz, 2H), 3.78 (s, 2H), 3.10 (s, 3H).^{13}C NMR (151 MHz, $CDCl_3$): δ 175.95, 167.23, 163.79, 137.92, 130.30, 77.16, 40.75, 21.10. HR-MS (m/z): Calcd for $C_{11}H_{10}N_4O_2$. Exact Mass: 230.0804, found: 231.0845 $[M+H]^+$.

Synthesis of Compound 2. Compound 1 (0.5 g, 2.2 mmol) and DMAP (0.5 g, 0.4 mmol) were dissolved in anhydrous DMF (10 mL) in a 100 mL three-necked round-bottom flask. Upon complete dissolution, EDCI (0.74 mg, 4.3 mmol) was added to the reaction mixture. The solution was stirred at 25 ^{o}C under a nitrogen atmosphere for 3 h. Subsequently, tert-butyl (6-aminohexyl) carbamate (0.47 g, 2.2 mmol) was introduced, and the reaction was allowed to proceed for an additional 3 h at 25 ^{o}C under continuous stirring. Upon completion, the reaction was quenched by the addition of deionized water (50 mL), and the resulting mixture was extracted with DCM (3×50 mL). The combined organic phases were sequentially washed with deionized water (3×50 mL) and saturated brine (3×50 mL), then dried over anhydrous Na_2SO_4. After filtration, the solvent was removed under reduced pressure to afford the crude product. Purification by silicone column chromatography (PE/DCM, 2:1, v/v) yielded compound 2 (0.54 g, 58%) as a purple solid. ^{1}H NMR (600 MHz, $CDCl_3$): δ 8.59 (d, J = 8.3 Hz, 2H), 7.50 (d, J = 8.4 Hz, 2H), 5.35 (s, 1H), 3.90 (s, 1H), 3.66 (s, 2H), 3.11 (s, 3H), 1.98 (t, J = 15.6 Hz, 2H), 1.70 (dd, J = 8.9, 4.2 Hz, 4H), 1.41 (s, 9H), 1.36 – 1.08 (m, 6H). ^{13}C NMR (151 MHz, $CDCl_3$): δ 169.21, 167.37, 163.83, 139.84, 130.99, 130.24, 128.56, 43.97, 31.94, 28.70, 28.40, 28.06, 21.19. HR-MS (m/z): Calcd for $C_{22}H_{32}N_6O_3$. Exact Mass: 428.2536, found: 429.2507 $[M+H]^+$.

Synthesis of Compound 3. Compound 2 (0.5 g mg, 1.17 mmol) was dissolved in anhydrous DCM (2 mL). TFA (174 μL) was added dropwise at 0 ^{o}C. The mixture was stirred at room temperature for 1 h, then concentrated under reduced pressure to obtain the deprotected product as a purple solid (420 mg, 84%). ^{1}H NMR (600 MHz, $CDCl_3$): δ 8.58 (d, J = 8.3 Hz, 2H), 7.50 (d, J = 8.3 Hz, 2H), 5.46 (s, 1H), 3.66 (s, 2H), 3.24 (d, J = 6.2 Hz, 2H), 3.10 (s, 3H), 2.64 (d, J = 7.0 Hz, 2H), 1.45 (d, J = 7.1 Hz, 2H), 1.40 (d, J = 6.9 Hz, 2H), 1.31 – 1.26 (m, 6H). ^{13}C NMR (151 MHz, $CDCl_3$): δ 169.93, 167.35, 163.87, 139.91, 132.57, 130.31, 128.50, 43.85, 42.04, 39.73, 33.55, 29.71, 26.61, 21.19. HR-MS (m/z): Calcd for $C_{17}H_{24}N_6O$. Exact Mass: 328.2012, found: 329.2034 $[M+H]^+$.

Synthesis of Compound 4. In a 25 mL round-bottom flask, PDSA (68.29 mg, 0.31 mmol) was dissolved in anhydrous DCM (5 mL). EDCI (104 mg, 0.54 mmol) was added under nitrogen and stirred for 5 min, followed by the addition of NHS (36 mg, 0.31 mmol). After 30 min, compound 3 (100 mg, 0.31 mmol) was added. The reaction was stirred at ambient temperature for 8 h. The mixture was extracted with DCM (3×20 mL), and the combined organic phases were washed with saturated brine (20 mL). The organic layer was dried over anhydrous Na_2SO_4, filtered, and

concentrated under reduced pressure to afford the crude product. Final purification was achieved by column chromatography using a solvent system of DCM/MeOH (95:5, v/v) containing 0.5% acetic acid as eluent, yielding 45 mg of the compound 4 (62 mg, 38.8%) as a purple solid. ^{1}H NMR (600 MHz, $CDCl_3$): *δ* 8.58 (d, J = 8.2 Hz, 2H), 7.50 (d, J = 8.2 Hz, 2H), 6.85 (s, 1H), 5.83 (s, 1H), 3.71 (s, 2H), 3.46 (s, 2H), 3.41 (s, 2H), 3.28 – 3.25 (m, 2H), 3.22 (d, J = 6.2 Hz, 2H), 3.11 (s, 3H), 1.61 (s, 6H), 1.49 (q, J = 6.6 Hz, 4H), 1.33 – 1.28 (m, 4H). ^{13}C NMR (151 MHz, $CDCl_3$): *δ* 172.23, 171.61, 169.19, 167.39, 163.81, 139.26, 131.12, 130.40, 128.60, 57.48, 43.60, 39.47, 35.06, 33.11, 30.15, 28.62, 25.35, 21.20. HR-MS (m/z): Calcd for $C_{24}H_{34}N_6O_4S_2$. Exact Mass: 534.2083, found: 535.1907 $[M+H]^+$.

Synthesis of Tz-tk-PEG. Compound 4 (40 mg, 0.08 mmol) and TEA (400 μL) were dissolved in DMSO (10 mL) in a 25 mL single-necked round-bottom flask. Upon complete dissolution, EDCI (30.7 mg, 0.16 mmol) was added to the reaction mixture. The solution was stirred at 25 ^{o}C under a nitrogen atmosphere for 1 h. Subsequently, MPEG2000-NH_2 (138 mg, 0.07 mmol) was introduced, and the reaction was allowed to proceed for an additional 3 h at 25 ^{o}C under continuous stirring. The reaction was quenched by the addition of 10 mL deionized water. The mixture was extracted with deionized water (3×10 mL), and the combined aqueous phases were washed with DCM (3×10 mL). The aqueous layer was dialyzed in fresh deionized water for 24 h (MWCO=2000 Da). Then, the solution was freeze-dried to afford the product Tz-tk-PEG (74 mg, 37.2%) as a purple solid. ^{1}H NMR (600 MHz, D_2O): *δ* 8.37 (s, 2H), 7.59 (s, 2H), 3.69 (s, 176H), 3.65 (s, 2H), 3.37 (s, 3H), 3.36 (s, 2H), 3.31 (s, 2H), 3.20 (m, 2H), 3.07 (s, 2H), 2.87 (s, 3H), 1.54 (s, 6H), 1.49 - 1.39 (m, 4H), 1.24 (s, 4H). HR-MS (m/z): Calcd for $C_{24}H_{33}N_6O_3S_2NH(C_2H_4O)_n$. Found: 2178.2204 - 2839.6283 $[M-H]^-$.

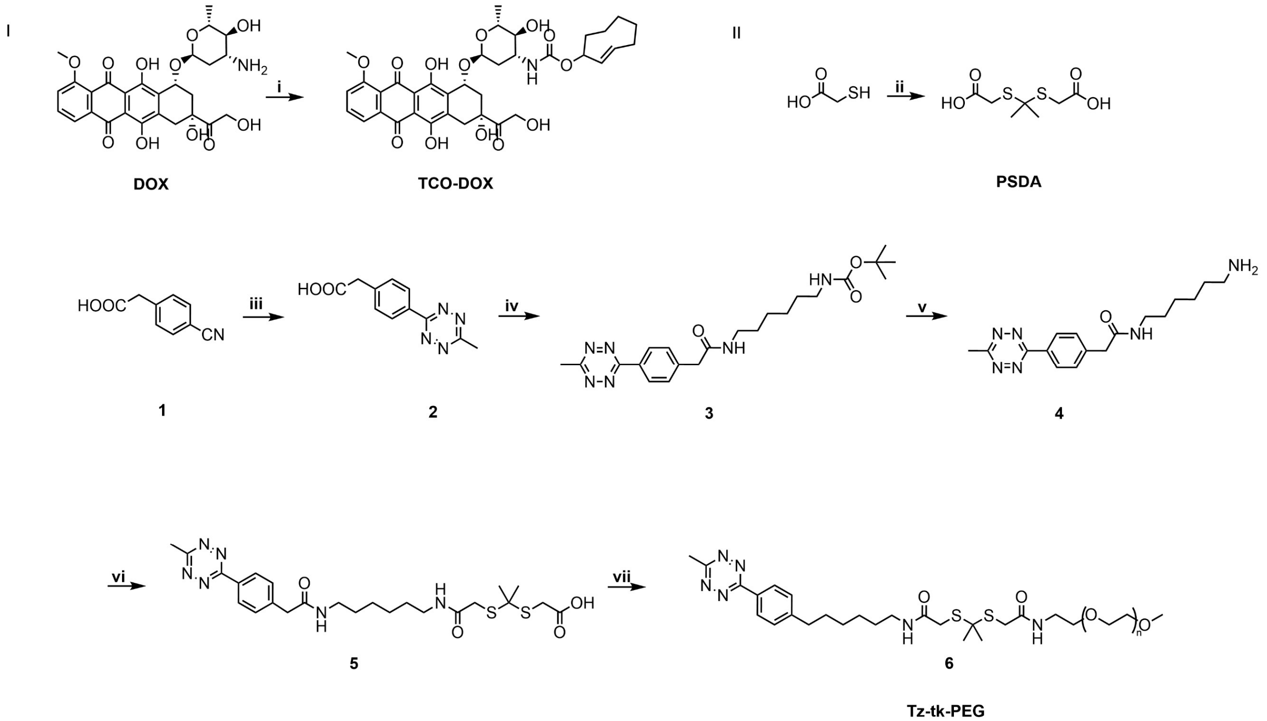

Figure S1. Synthetic route of TCO-DOX and Tz-tk-PEG. i) (E)-Cyclooct-2-en-1-yl (4-nitrophenyl) carbonate, DIEA, DMF, 30 °C, 24 h, 53.3%; ii) TFA, acetone, 25 °C, 3 h, 82.1%; iii) $Zn(OTf)_2$, hydrazine hydrate, MeCN, 60 °C, 24 h, 35.0%; iv) tert-butyl (6-aminohexyl) carbamate, EDCl, DMAP, 25 °C, 3 h, 58.0%; v) TFA, DCM, 1 h, 84%; vii) PSDA, EDCl, NHS, 8 h, 68.0%; vii) MPEG2000-NH_2, TEA, DMSO, 25 °C, 8 h, 37.2%.

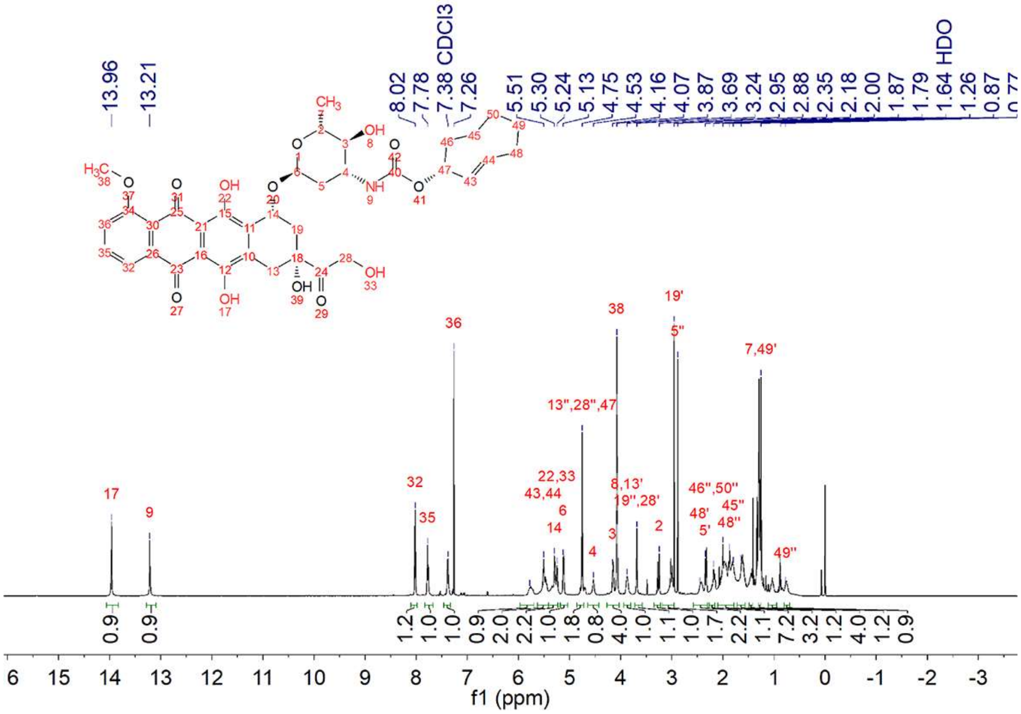

Figure S2. ^1H NMR analysis of TCO-DOX in $CDCl_3$ (600 MHz).

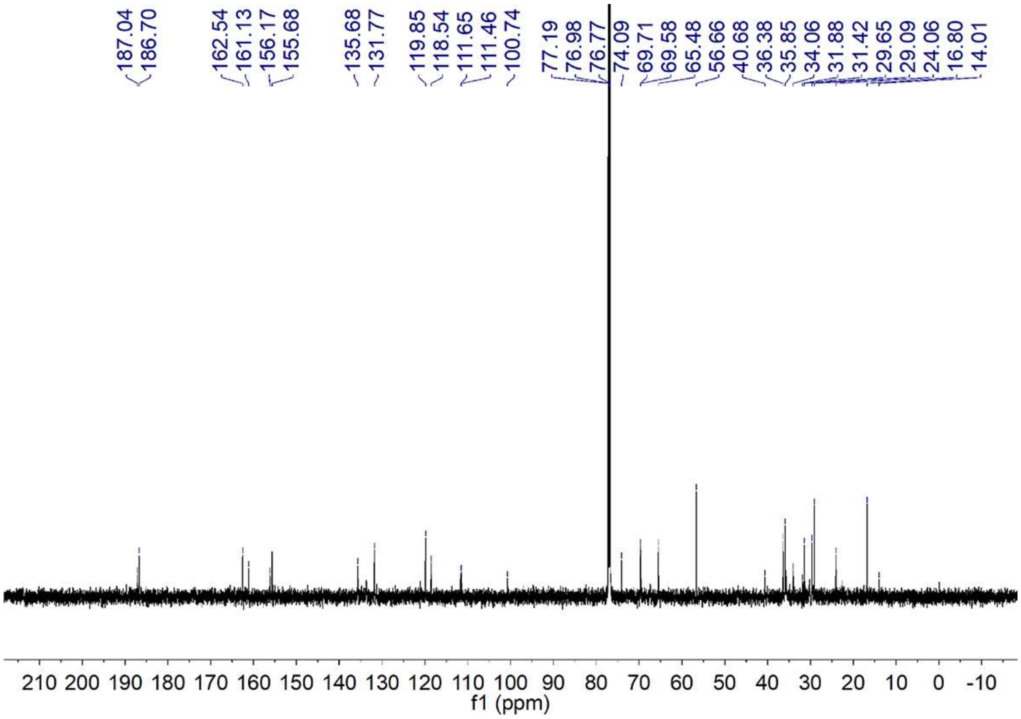

Figure S3. ^{13}C NMR analysis of TCO-DOX in $CDCl_3$ (151 MHz).

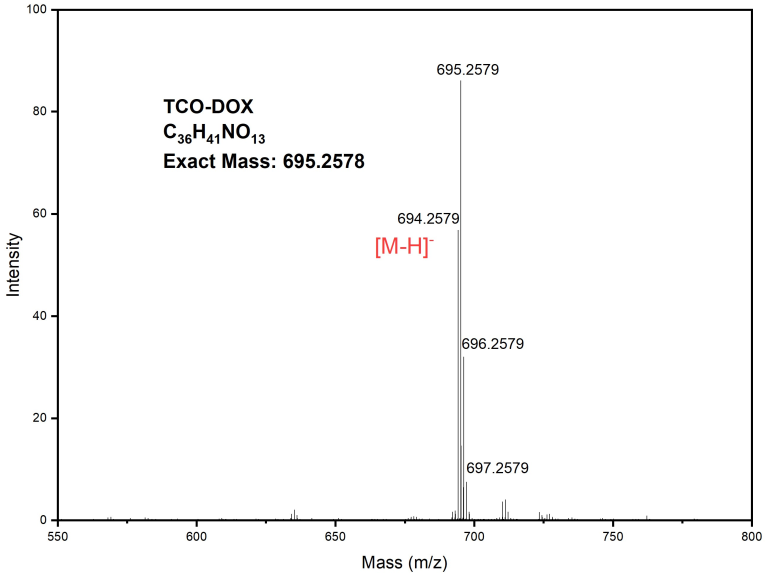

Figure S4. HR-MS analysis of compound TCO-DOX.

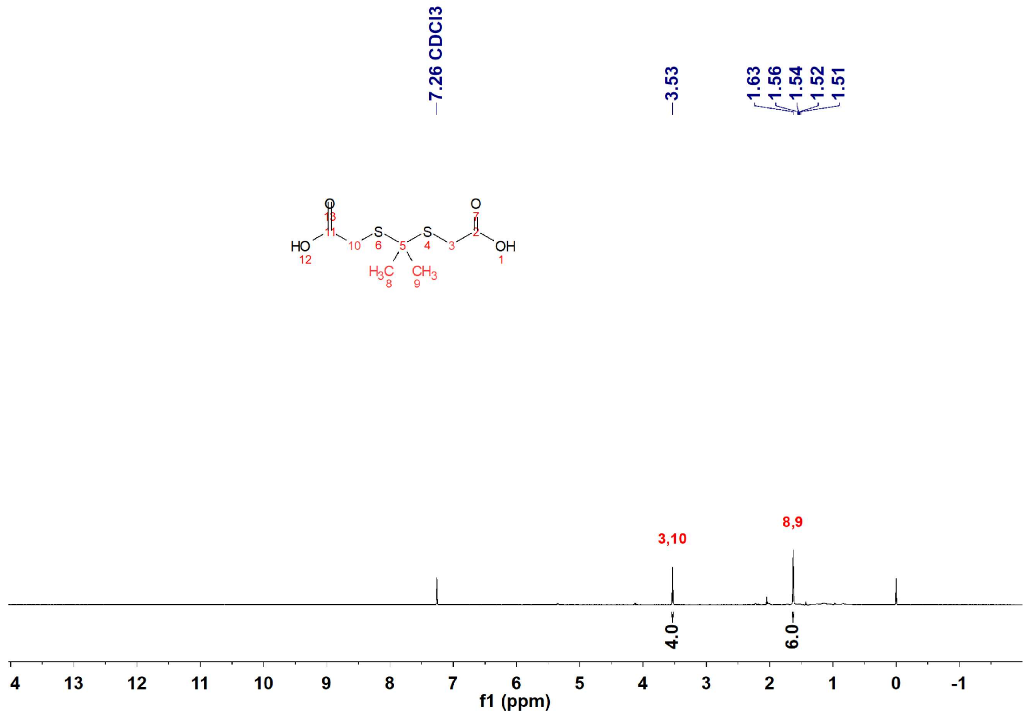

Figure S5. ^{1}H NMR analysis of PSDA in $CDCl_3$ (400 MHz).

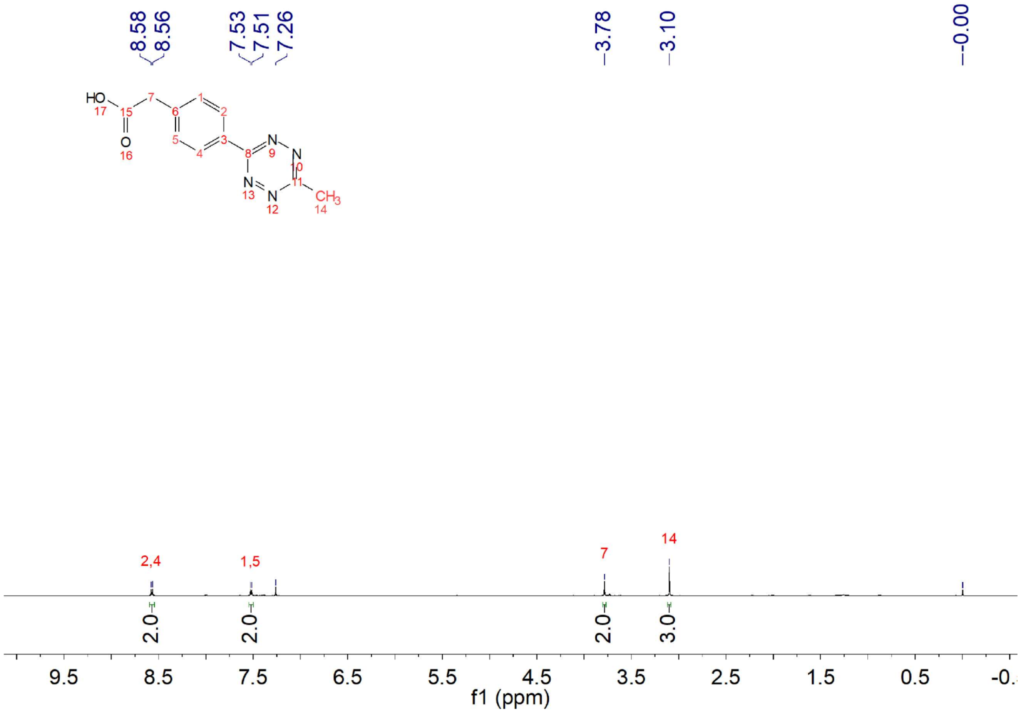

Figure S6. ^{1}H NMR analysis of compound 1 in $CDCl_3$ (600 MHz).

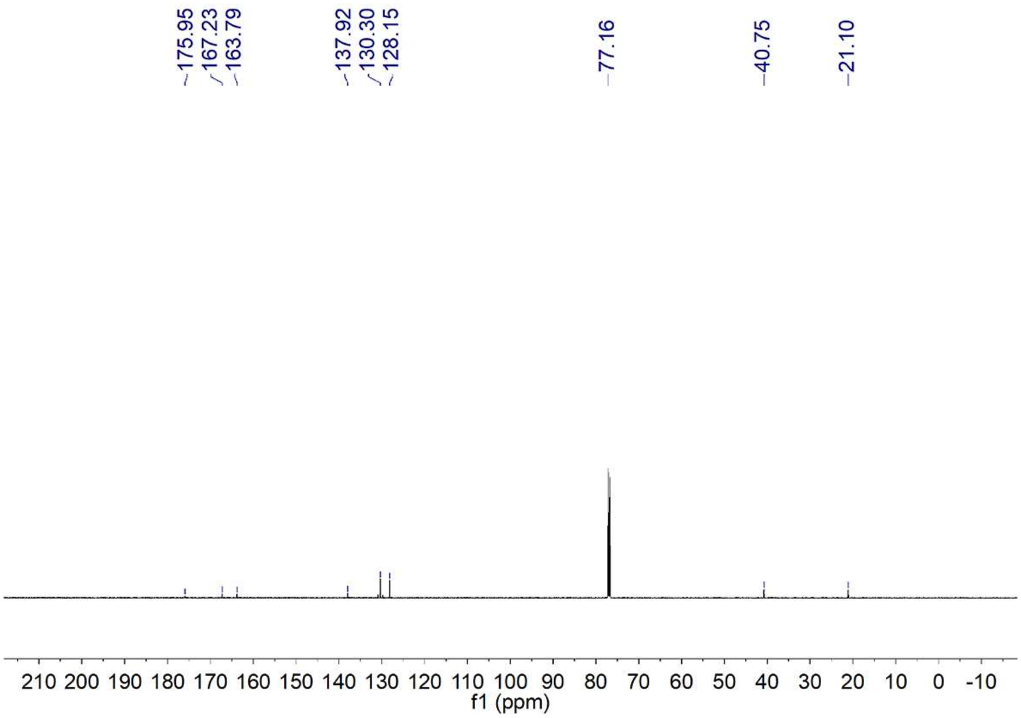

Figure S7. ^{13}C NMR analysis of compound 1 in $CDCl_3$ (151 MHz).

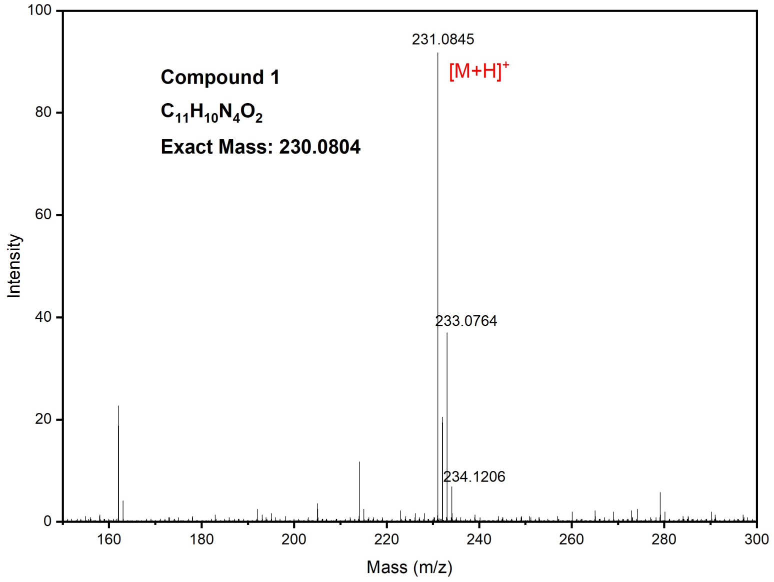

Figure S8. HR-MS analysis of compound 1.

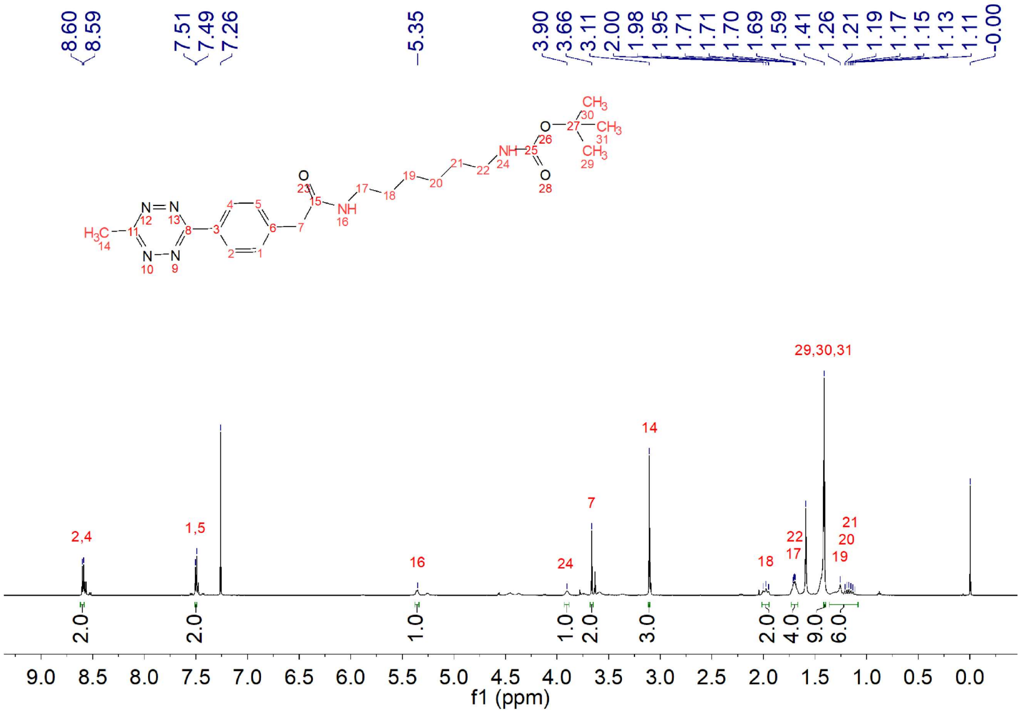

Figure S9. ^{1}H NMR analysis of compound 2 in $CDCl_3$ (600 MHz).

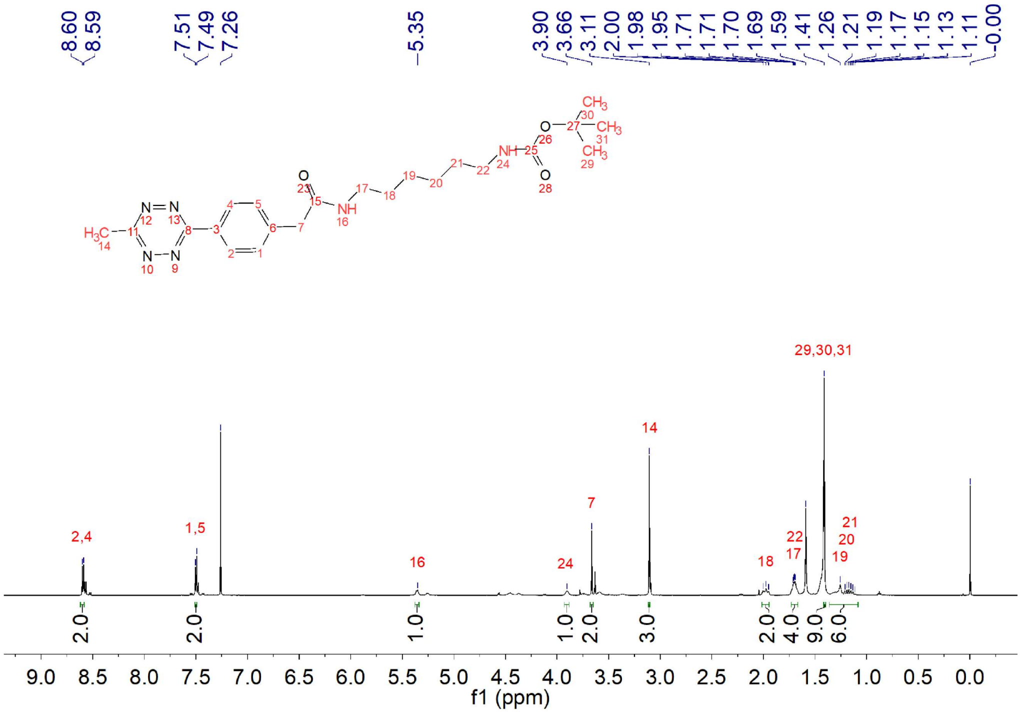

Figure S10. ^{13}C NMR analysis of compound 2 in $CDCl_3$ (151 MHz).

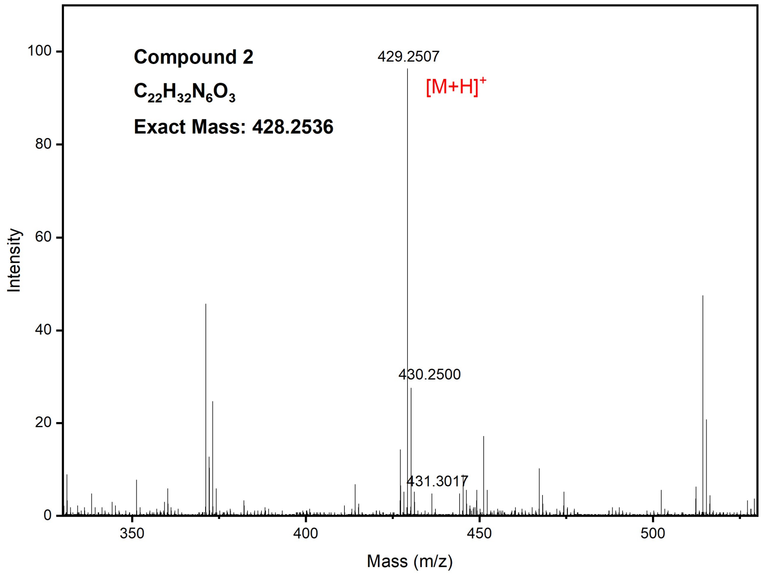

Figure S11. HR-MS analysis of compound 2.

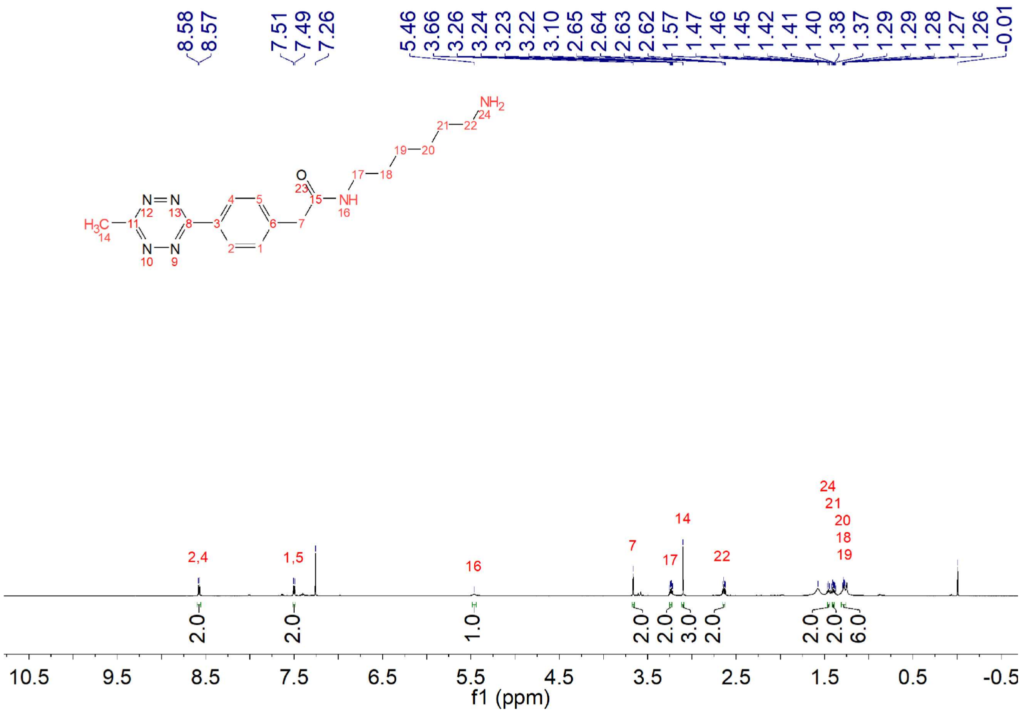

Figure S12. ^{1}H NMR analysis of compound 3 in $CDCl_3$ (600 MHz).

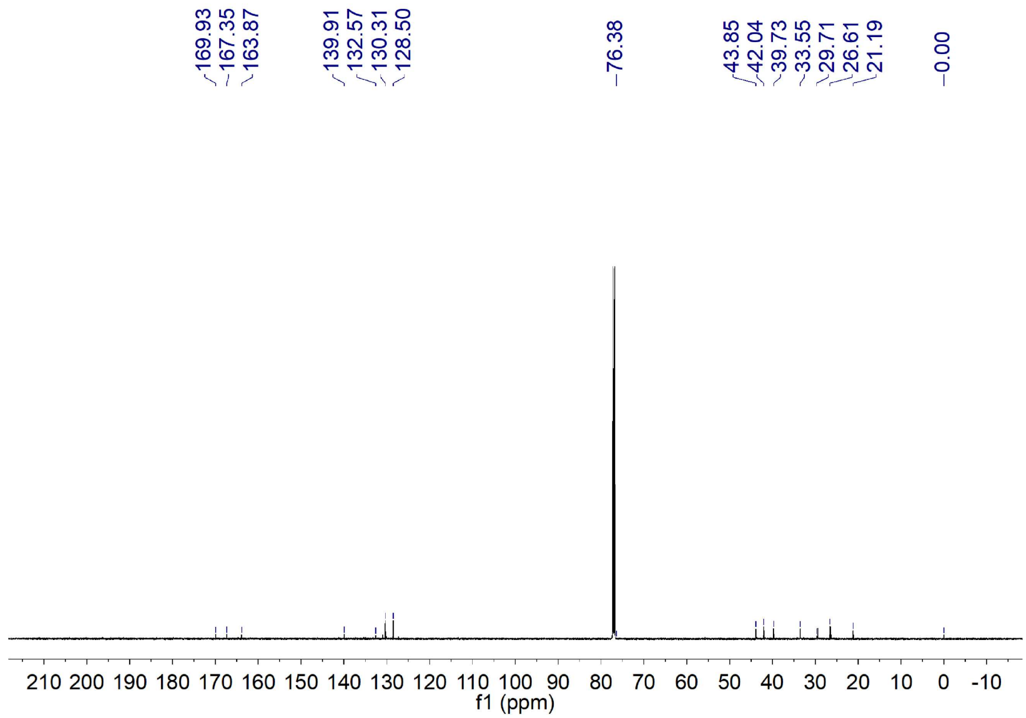

Figure S13. ^{13}C NMR analysis of compound 3 in $CDCl_3$ (151 MHz).

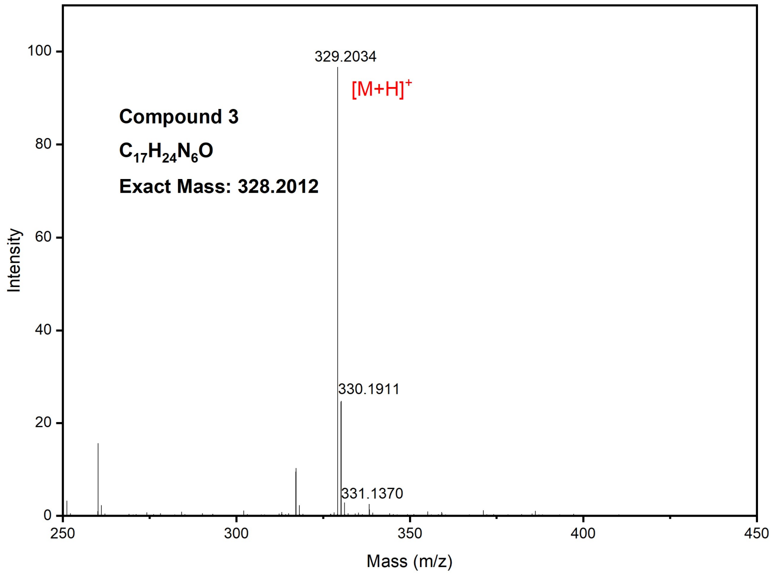

Figure S14. HR-MS analysis of compound 3.

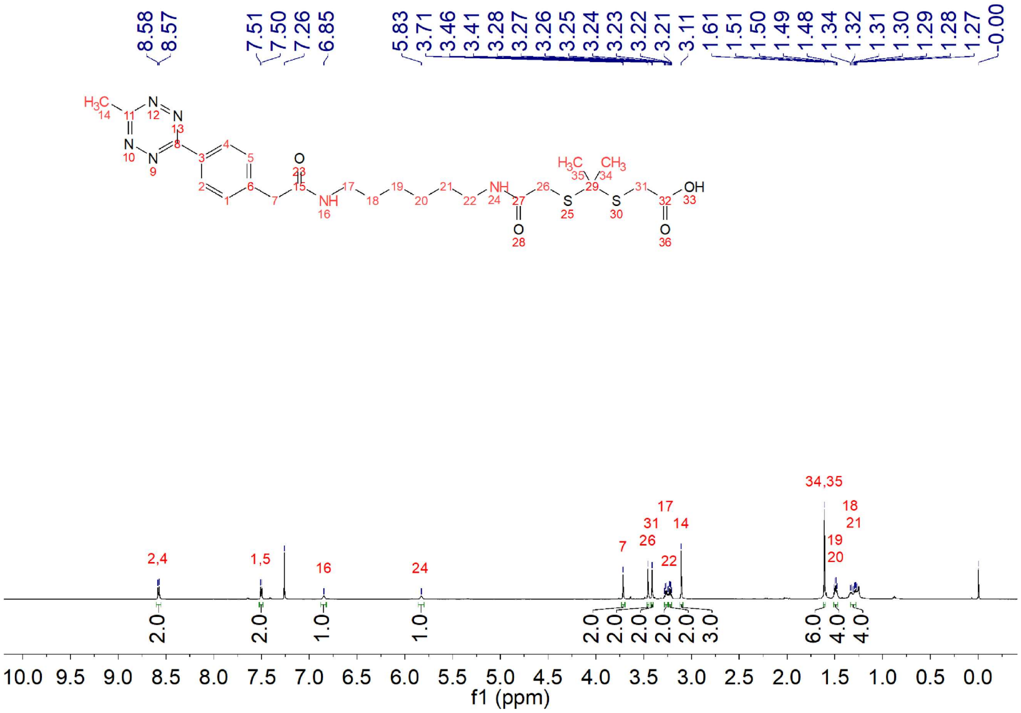

Figure S15. ^{1}H NMR analysis of compound 4 in $CDCl_3$ (600 MHz).

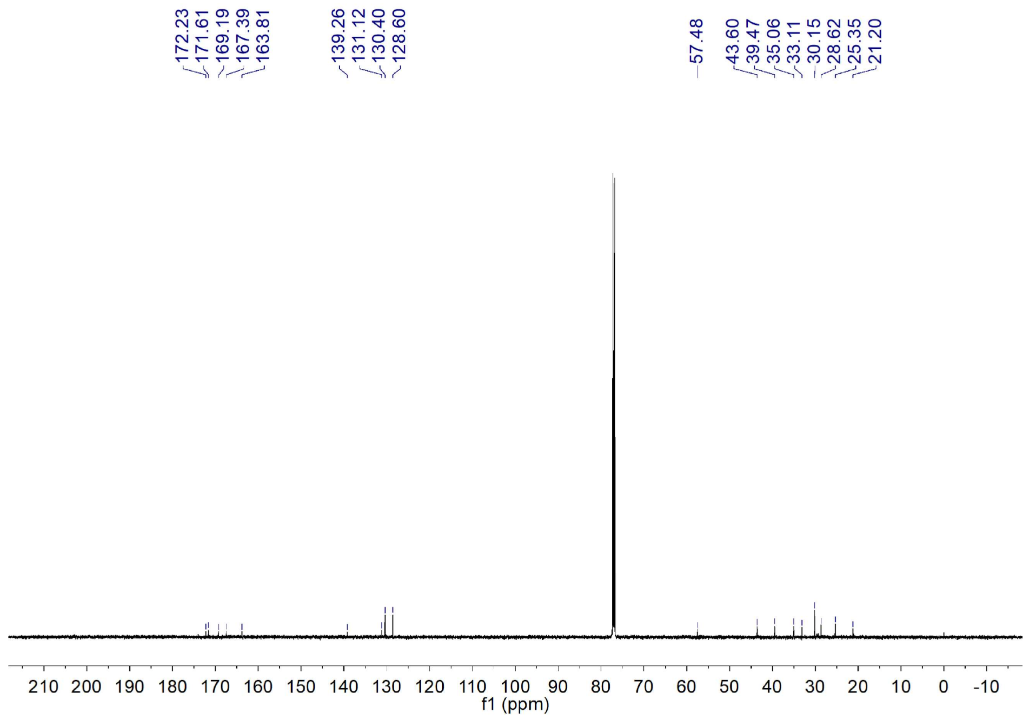

Figure S16. ^{13}C NMR analysis of compound 4 in $CDCl_3$ (151 MHz).

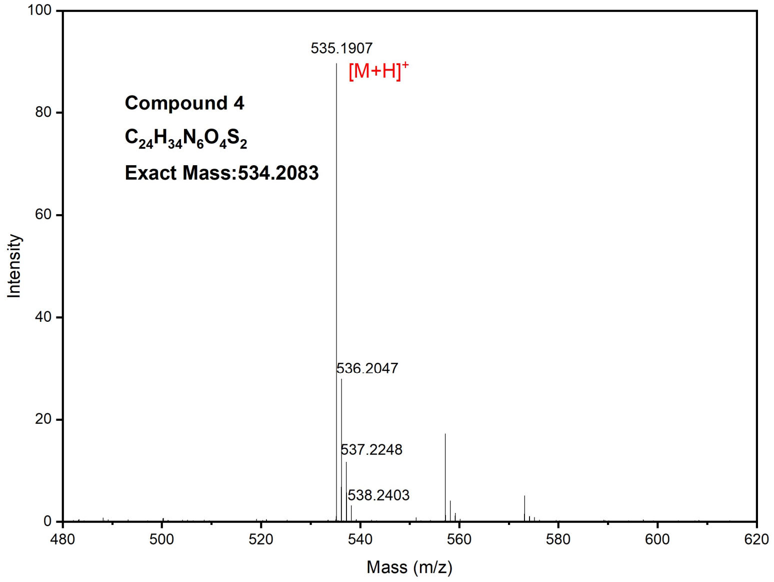

Figure S17. HR-MS analysis of compound 4.

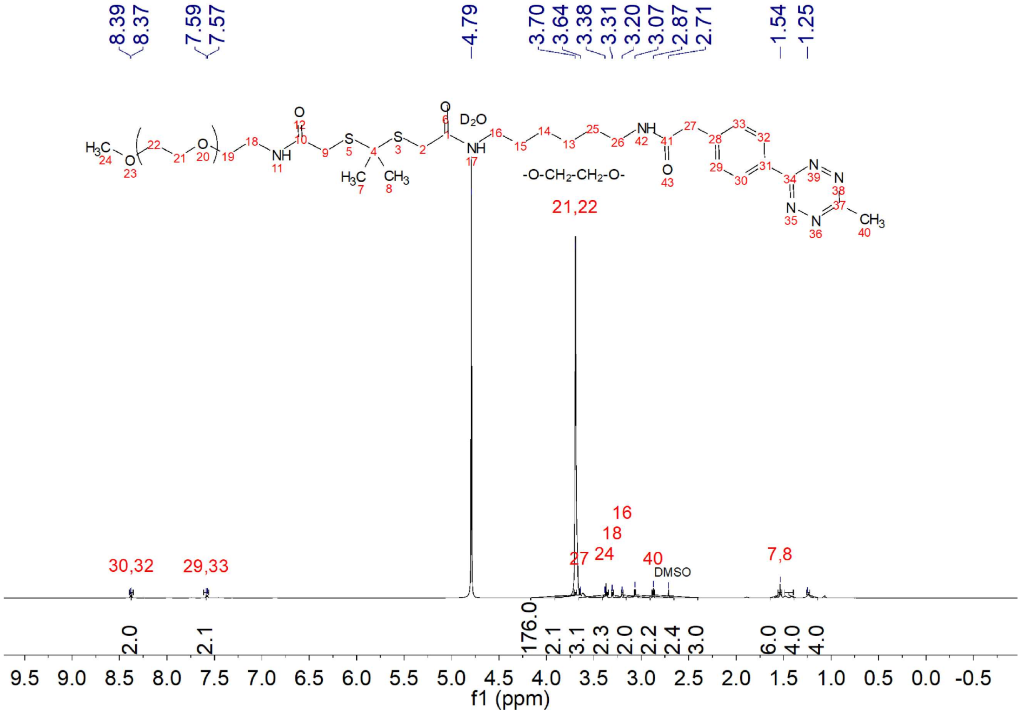

Figure S18. ^{1}H NMR analysis of Tz-tk-PEG in D_2O (600 MHz).

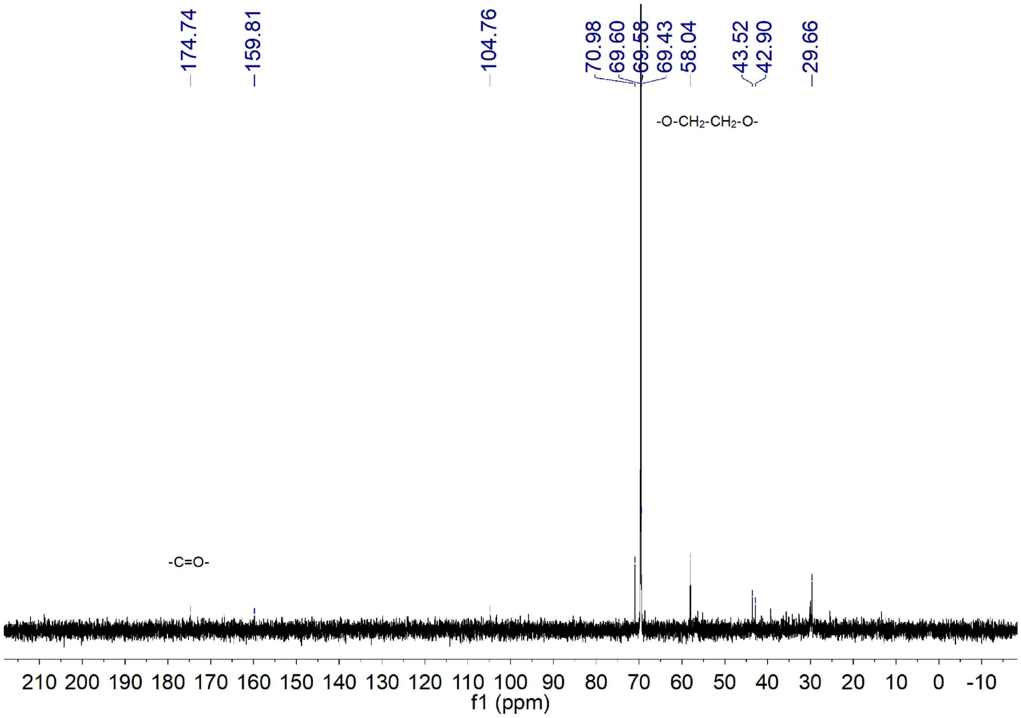

Figure S19. ^{13}C NMR analysis of Tz-tk-PEG in D_2O (151 MHz).

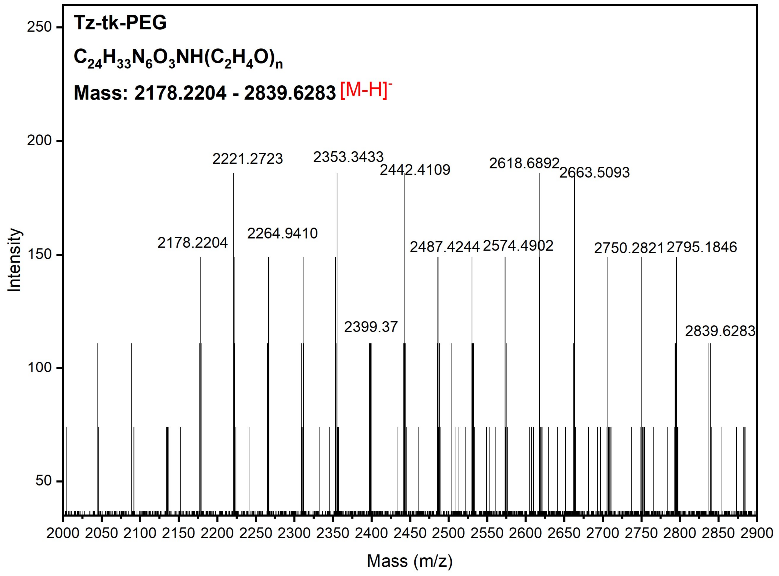

Figure S20. HR-MS analysis of Tz-tk-PEG.

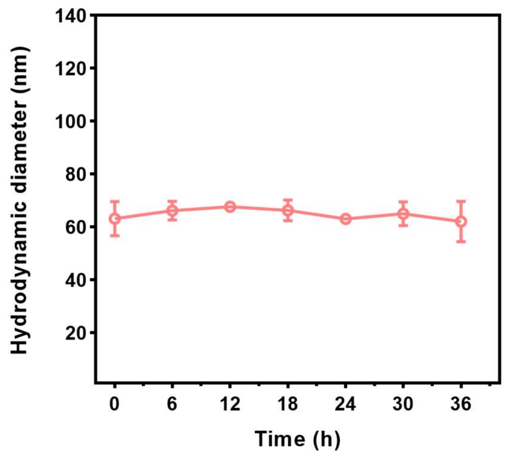

Figure S21. Size variation of TCO-DOX@ZIF-8 in PBS over 36 hours (n = 3 independent experiments). Data points were presented as mean ± s.d..

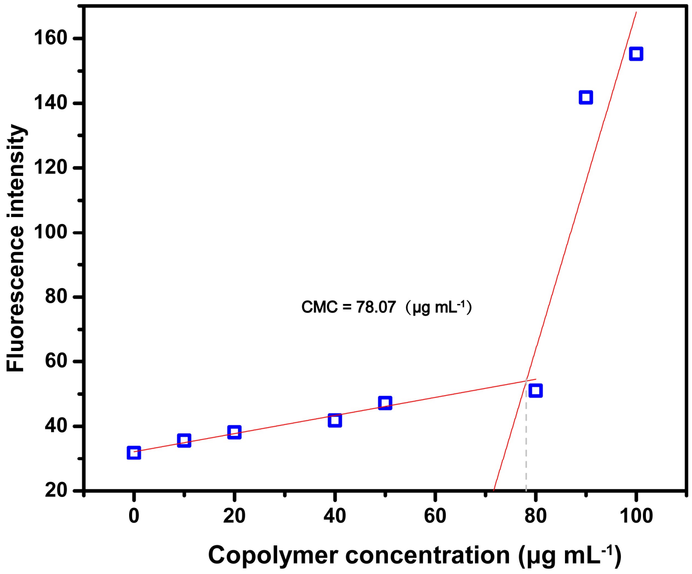

Figure S22. Representative fluorescence data for CMC calculation: Nile Red fluorescence intensity versus Tz-tk-PEG concentration.

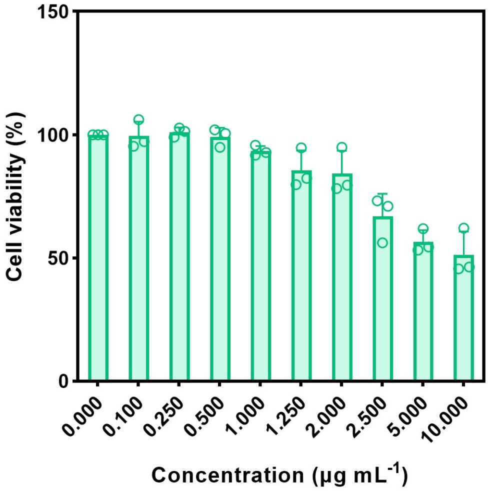

Figure S23. Cell viability of 4T1 cells treated with ICG at different concentrations under laser irradiation (n = 3 independent experiments). Data points were presented as mean ± s.d..

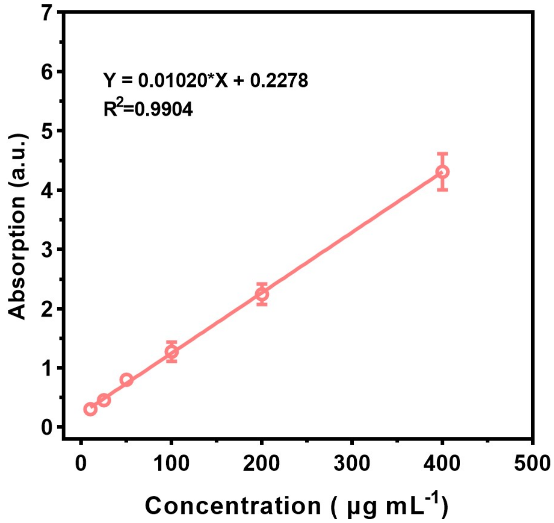

Figure S24. The calibration curve of ICG determined through UV-Vis spectrum analysis (n = 3 independent experiments). Data points were presented as mean ± s.d..

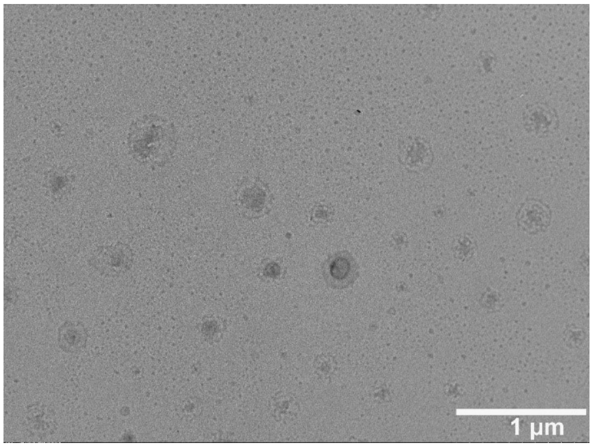

Figure S25. The TEM image of ICG@Tz-tk-PEG following irradiation with an 808 nm laser (0.8 W cm^{-2}, 2 min). Scale bar = 1 μm.

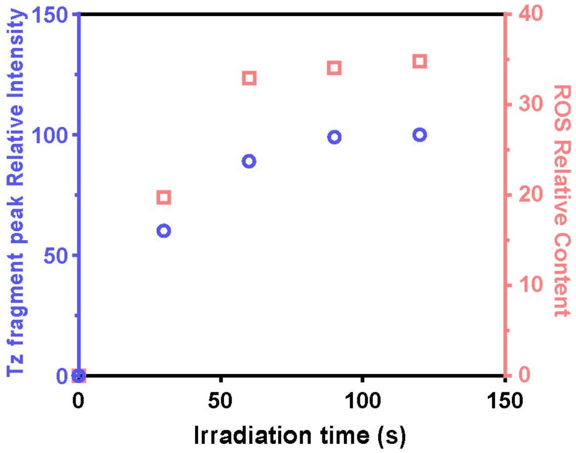

Figure S26. Correlation between ROS relative content and Tz fragment peak intensity over irradiation time.

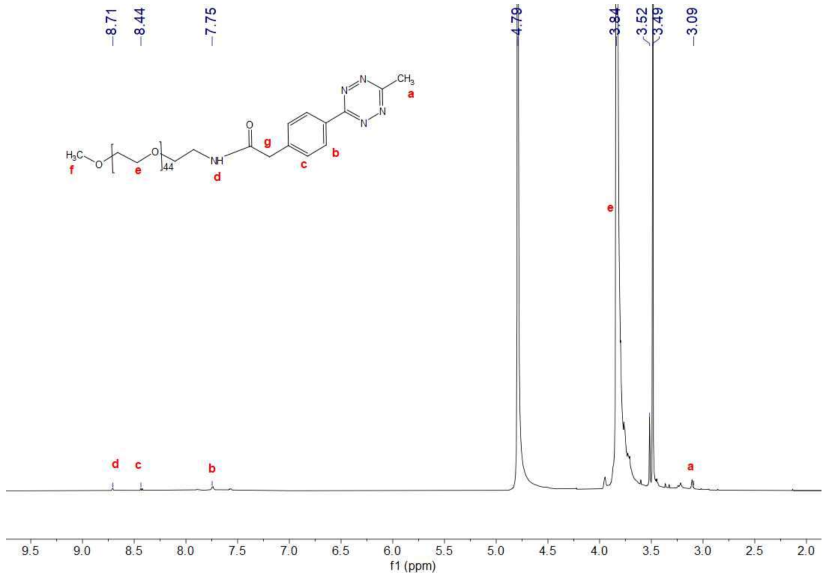

Figure S27. Chemical structures of PEG-Tz and ^{1}H NMR analysis of PEG-Tz in $CDCl_3$ (600 MHz).

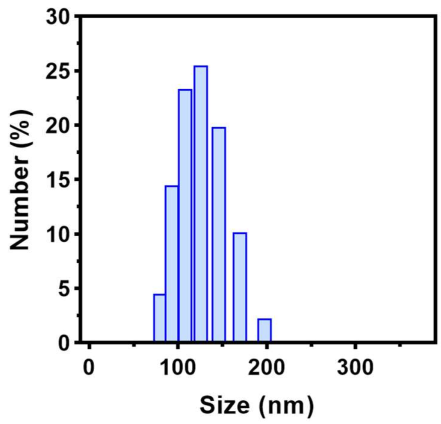

Figure S28. Size distribution of ICG@Tz-PEG.

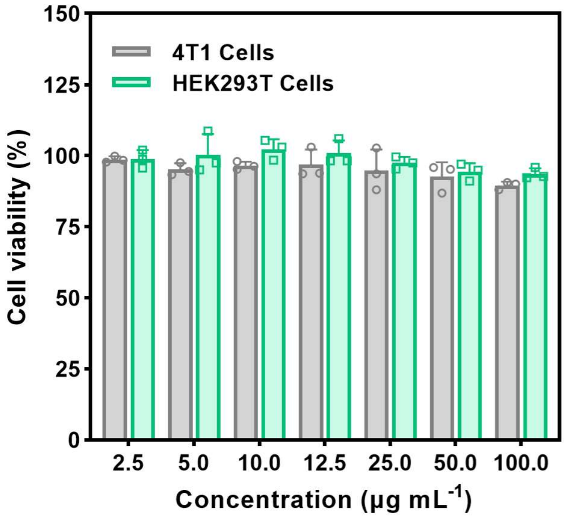

Figure S29. Viability of 4T1 tumor and HEK293T normal liver cells separately treated with TCO-DOX@ZIF-8 at different concentrations (n = 3 independent experiments). Data points were presented as mean ± s.d..

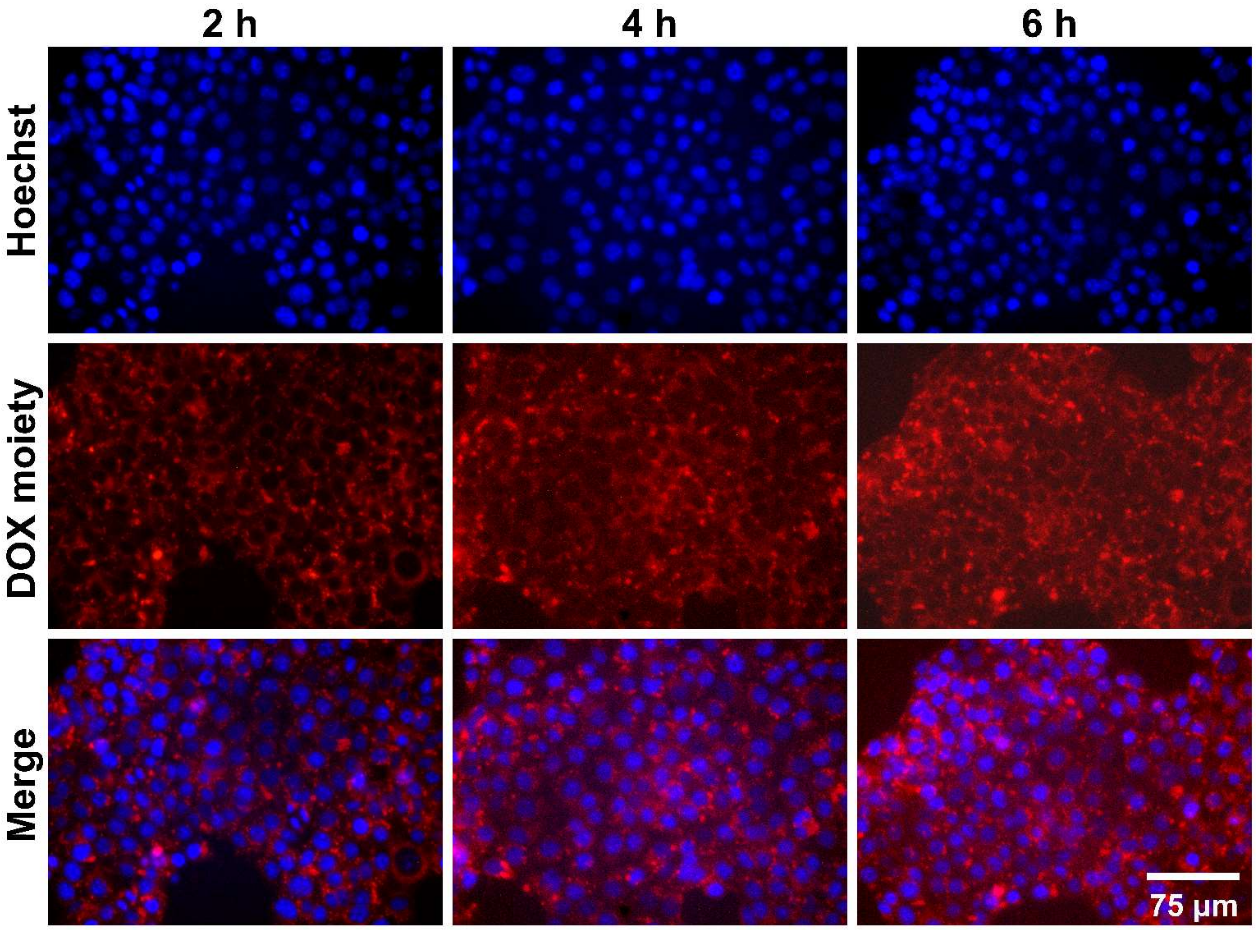

Figure S30. Time-dependent cellular uptake of TCO-DOX@ZIF-8 by 4T1 cells, as visualized by fluorescence microscopy. Scale bar = 75 μm.

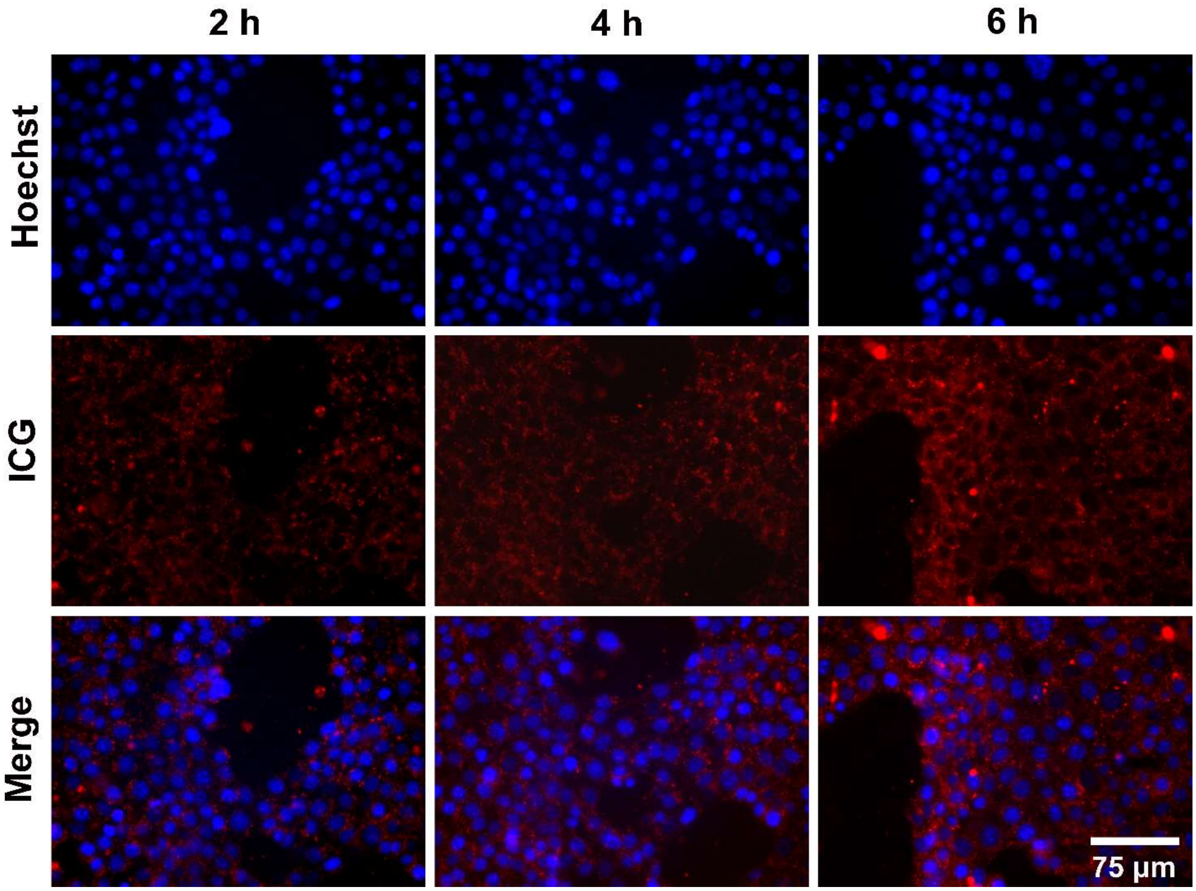

Figure S31. Time-dependent cellular uptake of ICG@Tz-tk-PEG by 4T1 cells, as visualized by fluorescence microscopy. Scale bar = 75 μm.

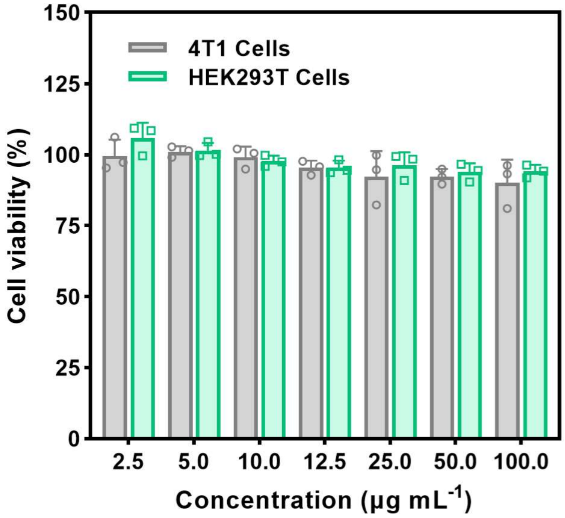

Figure S32. 4T1 and HEK293T cell viabilities after incubation with different concentrations of ICG@Tz-tk-PEG (n = 3 independent experiments). Data points were presented as mean ± s.d..

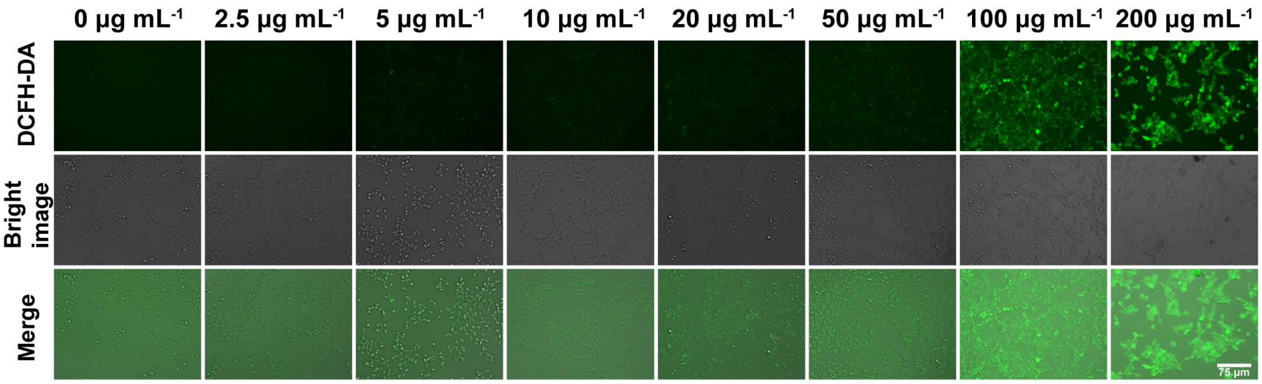

Figure S33. Detection of ROS generation by DCFH-DA in 4T1 cells after incubation with different concentrations of ICG@Tz-tk-PEG nanomicelles under 808 nm laser irradiation (0.8 W cm^{-2}, 2 min). Scale bar = 75 μm.

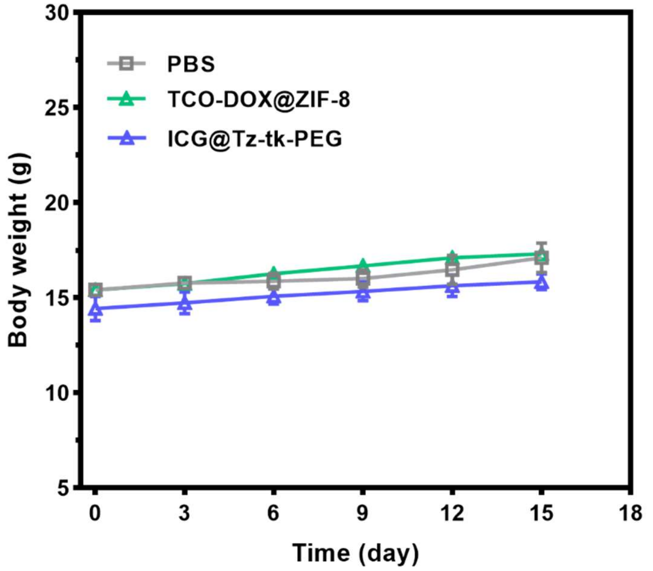

Figure S34. Variation of the mouse body weight over time in different groups (n = 3 mice per group). Data points were presented as mean ± s.d..

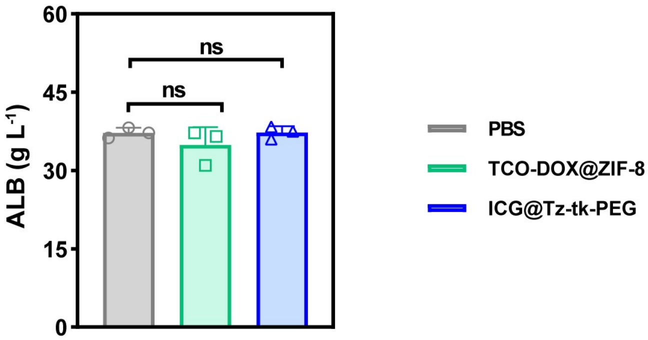

Figure S35. Biochemical analysis of liver injury indicators of mice with PBS, TCO-DOX@ZIF-8, or ICG@Tz-tk-PEG administration (n = 3 biologically independent experiments per group). Data points were presented as mean ± s.d.. Statistical analysis was performed using one-way ANOVA. “ns” represents no significant difference.

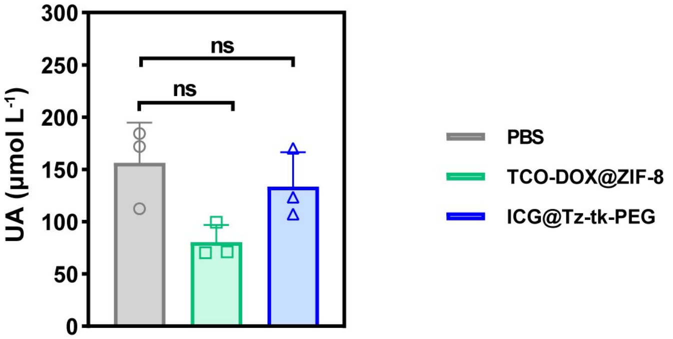

Figure S36. Biochemical analysis of kidney injury indicators of mice with PBS, TCO-DOX@ZIF-8, or ICG@Tz-tk-PEG administration (n = 3 mice per group). Data points were presented as mean ± s.d.. Statistical analysis was calculated via one-way ANOVA with a Tukey post-hoc test. “ns” represents no significant difference.

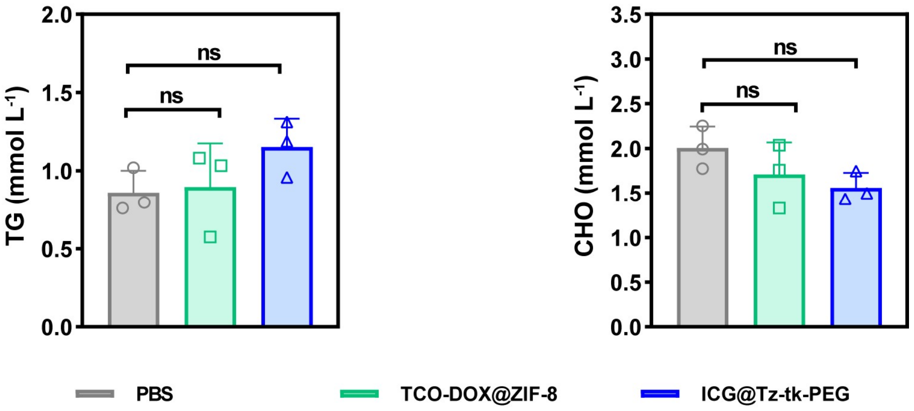

Figure S37. Biochemical analysis of lipids in mice with PBS, TCO-DOX@ZIF-8, or ICG@Tz-tk-PEG administration (n = 3 mice per group). Data points were presented as mean ± s.d.. Statistical analysis was calculated via one-way ANOVA with a Tukey post-hoc test. “ns” represents no significant difference.

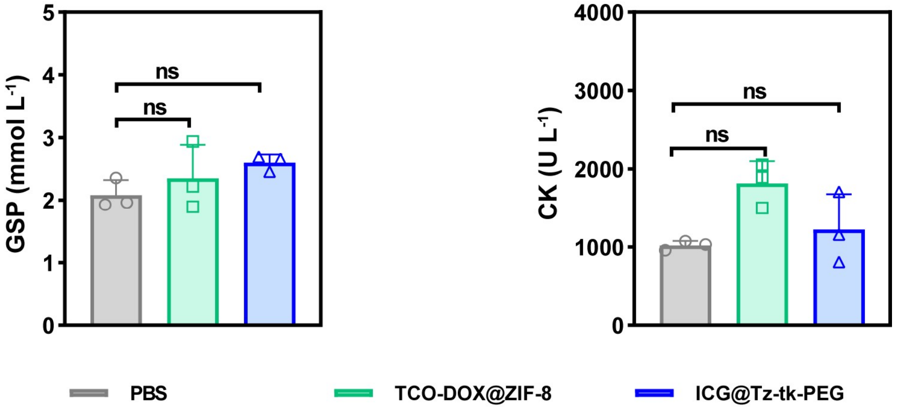

Figure S38. Biochemical analysis of blood glucose (left) and cardiac enzyme profiles (right) of mice with PBS, TCO-DOX@ZIF-8, or ICG@Tz-tk-PEG administration (n = 3 mice per group). Data points were presented as mean ± s.d.. Statistical analysis was calculated via one-way ANOVA with a Tukey post-hoc test. “ns” represents no significant difference.

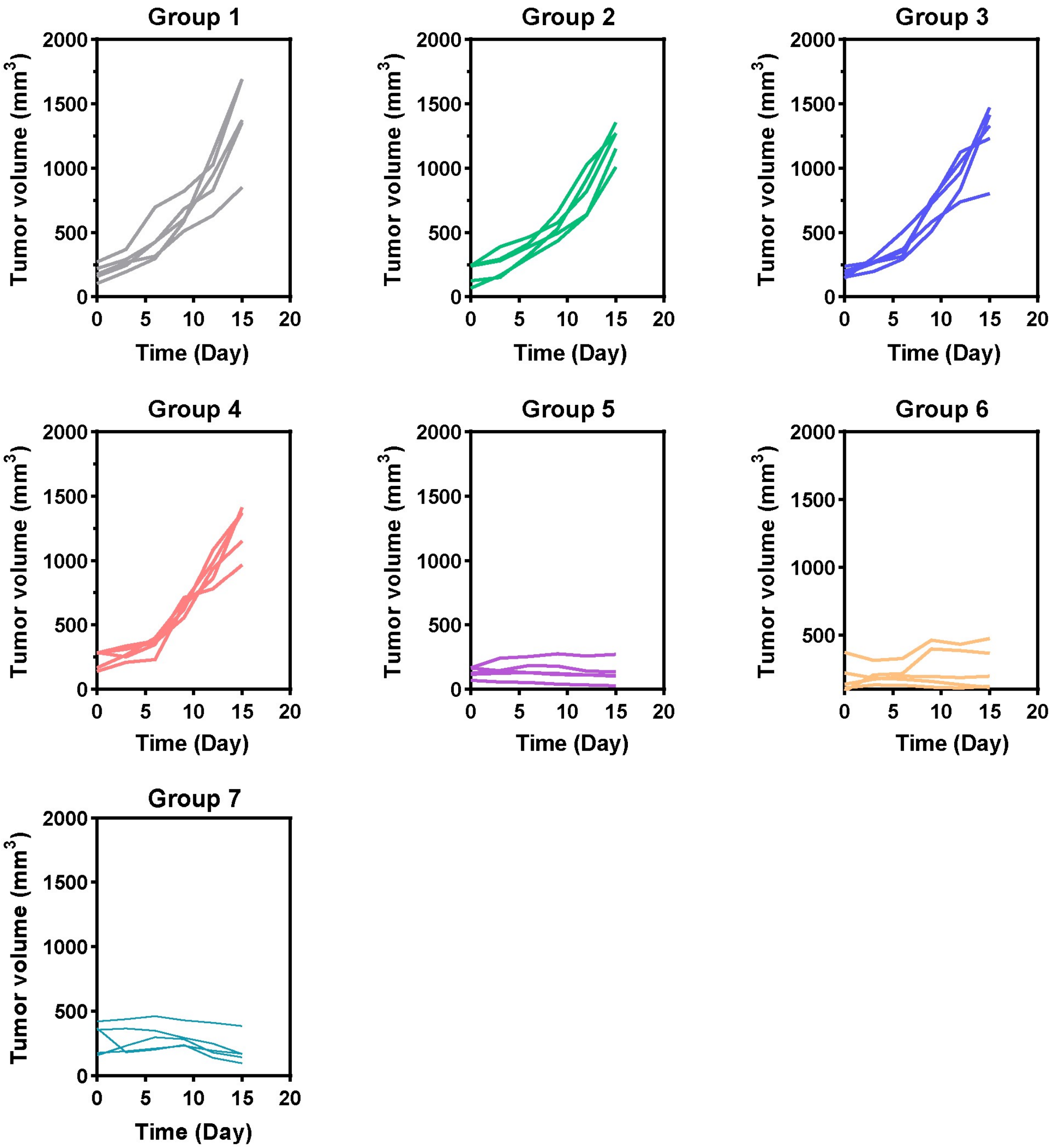

Figure S39. The growth curves of tumors in mice from different groups: Group 1, PBS; Group 2, TCO-DOX@ZIF-8; Group 3, ICG@Tz-tk-PEG + laser irradiation; Group 4, TCO-DOX@ZIF-8 + ICG@Tz-tk-PEG; Group 5, TCO-DOX@ZIF-8 + ICG@Tz-tk-PEG + laser irradiation; Group 6, free DOX; and Group 7, DOX@ZIF-8.
